# Therapeutic effects of sphingosine kinase inhibitor N,N-dimethylsphingosine (DMS) in experimental chronic Chagas disease cardiomyopathy

**DOI:** 10.1038/s41598-017-06275-z

**Published:** 2017-07-21

**Authors:** Juliana Fraga Vasconcelos, Cássio Santana Meira, Daniela Nascimento Silva, Carolina Kymie Vasques Nonaka, Pâmela Santana Daltro, Simone Garcia Macambira, Pablo Daniel Domizi, Valéria Matos Borges, Ricardo Ribeiro-dos-Santos, Bruno Solano de Freitas Souza, Milena Botelho Pereira Soares

**Affiliations:** 1 Instituto Gonçalo Moniz, Fundação Oswaldo Cruz, FIOCRUZ, Salvador, BA 40296-710 Brazil; 2 Centro de Biotecnologia e Terapia Celular, Hospital São Rafael, Salvador, BA 41253-190 Brazil; 3 Escola de Ciências da saúde, Universidade Salvador, Salvador, BA 41720-200 Brazil; 4 Departamento de Bioquímica e Biofísica, Instituto de Ciências da Saúde, Universidade Federal da Bahia, Salvador, BA 40110-100 Brazil; 5 Centro de Ciências da Saúde, Instituto de Biofísica Carlos Chagas Filho, Universidade Federal do Rio de Janeiro, Rio de Janeiro, RJ 21944-970 Brazil

## Abstract

Chagas disease cardiomyopathy is a parasite-driven inflammatory disease to which there are no effective treatments. Here we evaluated the therapeutic potential of N,N-dimethylsphingosine(DMS), which blocks the production of sphingosine-1-phosphate(S1P), a mediator of cellular events during inflammatory responses, in a model of chronic Chagas disease cardiomyopathy. DMS-treated, *Trypanosoma cruzi*-infected mice had a marked reduction of cardiac inflammation, fibrosis and galectin-3 expression when compared to controls. Serum concentrations of galectin-3, IFNγ and TNFα, as well as cardiac gene expression of inflammatory mediators were reduced after DMS treatment. The gene expression of M1 marker, iNOS, was decreased, while the M2 marker, arginase1, was increased. DMS-treated mice showed an improvement in exercise capacity. Moreover, DMS caused a reduction in parasite load *in vivo*. DMS inhibited the activation of lymphocytes, and reduced cytokines and NO production in activated macrophage cultures *in vitro*, while increasing IL-1β production. Analysis by qRT-PCR array showed that DMS treatment modulated inflammasome activation induced by *T. cruzi* on macrophages. Altogether, our results demonstrate that DMS, through anti-parasitic and immunomodulatory actions, can be beneficial in the treatment of chronic phase of *T. cruzi* infection and suggest that S1P-activated processes as possible therapeutic targets for the treatment of Chagas disease cardiomyopathy.

## Introduction

The pathological manifestations of chronic Chagas disease, caused by *Trypanosoma cruzi* infection, both in the cardiac and in the digestive form, are associated with the occurrence of an inflammatory reaction^1^. Chronic Chagas disease cardiomyopathy (CCC) involves cardiac myocytes undergoing necrosis and cytolysis via various mechanisms, and areas of myocellular hypertrophy and mononuclear cell infiltration occur^2–4^. In response to the myocardial damage, fibrotic areas occur and may contribute to the disruption of the cardiac conduction system and appearance of dysrhythmias, as well as to myocardial thinning and cardiac hypertrophy^5^. Given the lack of an effective specific therapy, CCC is treated similarly to all other heart failure syndromes using therapies to mitigate symptoms^6^. Therefore, the development of new alternative treatments for CCC is needed.

Sphingolipid metabolites are emerging as important lipid signaling molecules in both health and disease^7^. Among them, sphingosine-1-phosphate (S1P), produced by phosphorylation of sphingosine (Sph) by sphingosine kinases (SphK1 and SphK2) in response to various stimuli, plays important roles in several cellular processes, including cell growth and cell trafficking^8, 9^. The balance of Sph and S1P determines the progress of many diseases and there is evidence that sphingolipid metabolism and the expression of S1P receptors (S1PR1-5) are altered in inflammatory processes^10^. S1P drives the differentiation of different immune cell types, inducing changes in their functional phenotypes and regulating production of pro-inflammatory cytokines and eicosanoids. In particular, S1P has emerged as a central regulator of lymphocyte egress^11, 12^.

Due to the persistent inflammation found in CCC, which is a hallmark of the disease, and the critical role of S1P-activated pathways on the regulation of inflammation, we hypothesized that N,N-dimethylsphingosine (DMS), a pan SphK inhibitor, has a beneficial effect in chronic Chagas disease. Thus, in the present study we investigated the effects of DMS in a murine model of chronic Chagas disease cardiomyopathy, as well as its mechanisms of action on *in vitro* assays.

## Results

### Treatment with DMS reduces heart inflammation and fibrosis in *T. cruzi*-infected mice

**Figure 1:**
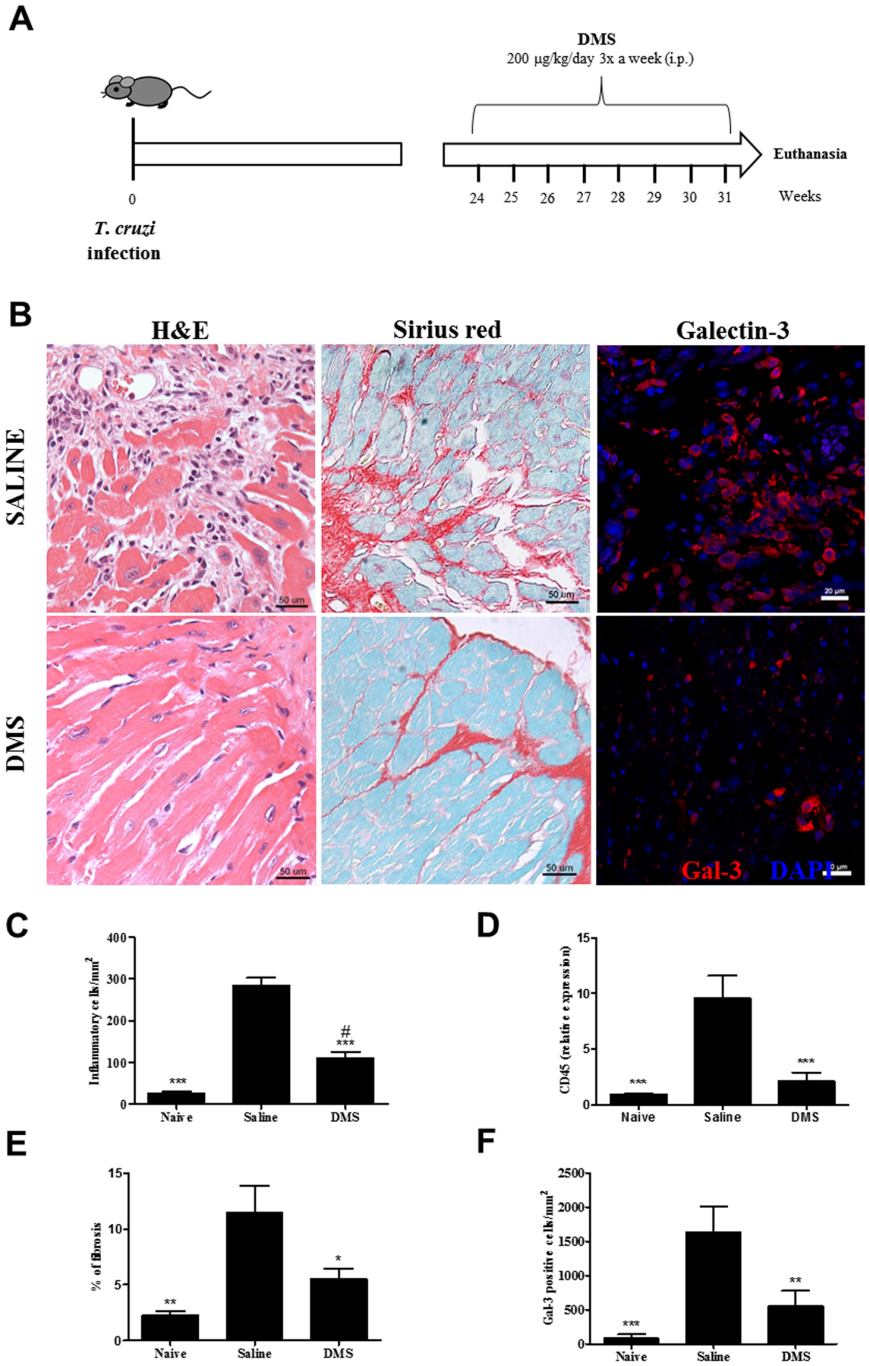
Reduction of inflammation, fibrosis and galectin-3 was found in the hearts of DMS-treated mice. (**A**) Experimental design of *in vivo* treatment. C57BL/6 mice infected with trypomastigotes (Colombian strain) were treated during the chronic phase of infection (6 months pos-infection) with DMS (200 µg/Kg/day; 3x week; i.p.). (**B**) Microphotographs of heart sections stained with hematoxylin and eosin or sirius red or anti-galectin-3 (1:50; red) and DAPI (blue). (**C**) Inflammatory cells were quantified in heart sections of naive mice, saline-treated chagasic mice, or DMS-treated chagasic mice and integrated by area. (**D**) The expression of CD45 was evaluated by real-time qRT-PCR using cDNA samples prepared from mRNA extracted from hearts of experimental groups. (**E**) Fibrotic area is represented by percentage of collagen deposition in heart sections. (**F**) Quantifications of galectin-3^+^ cells in heart sections were performed in ten random fields captured under 400x magnification, using the Image Pro Plus v.7.0 software. Bars represent means ± SEM of 10 mice/group. ***P < 0.001; **P < 0.01; *P < 0.05 compared to saline group; ^#^ *P* < 0.05 compared to naive group.

Groups of mice chronically infected with *T. cruzi* were treated with DMS or vehicle (saline) (Fig. 1A). Inflammation and fibrosis were evaluated in heart sections two months after the first dose. A diffuse inflammatory response, mainly composed of mononuclear cells, was found in saline-treated infected controls (Fig. 1B). Administration of DMS caused a marked reduction in the number of inflammatory cells, which was statistically significant when compared to vehicle-treated mice (Fig. 1B,C). Gene expression of CD45, a pan-leukocyte marker, which was increased in *T. cruzi* infected mice treated with saline, was also significantly reduced after DMS treatment (Fig. 1D). Similarly, heart sections from DMS-treated mice had a reduced percentage of fibrosis when compared with vehicle-treated mice (Fig. 1B,E).

### Galectin-3 reduction in the heart and sera of chagasic mice after DMS treatment

**Figure 2:**
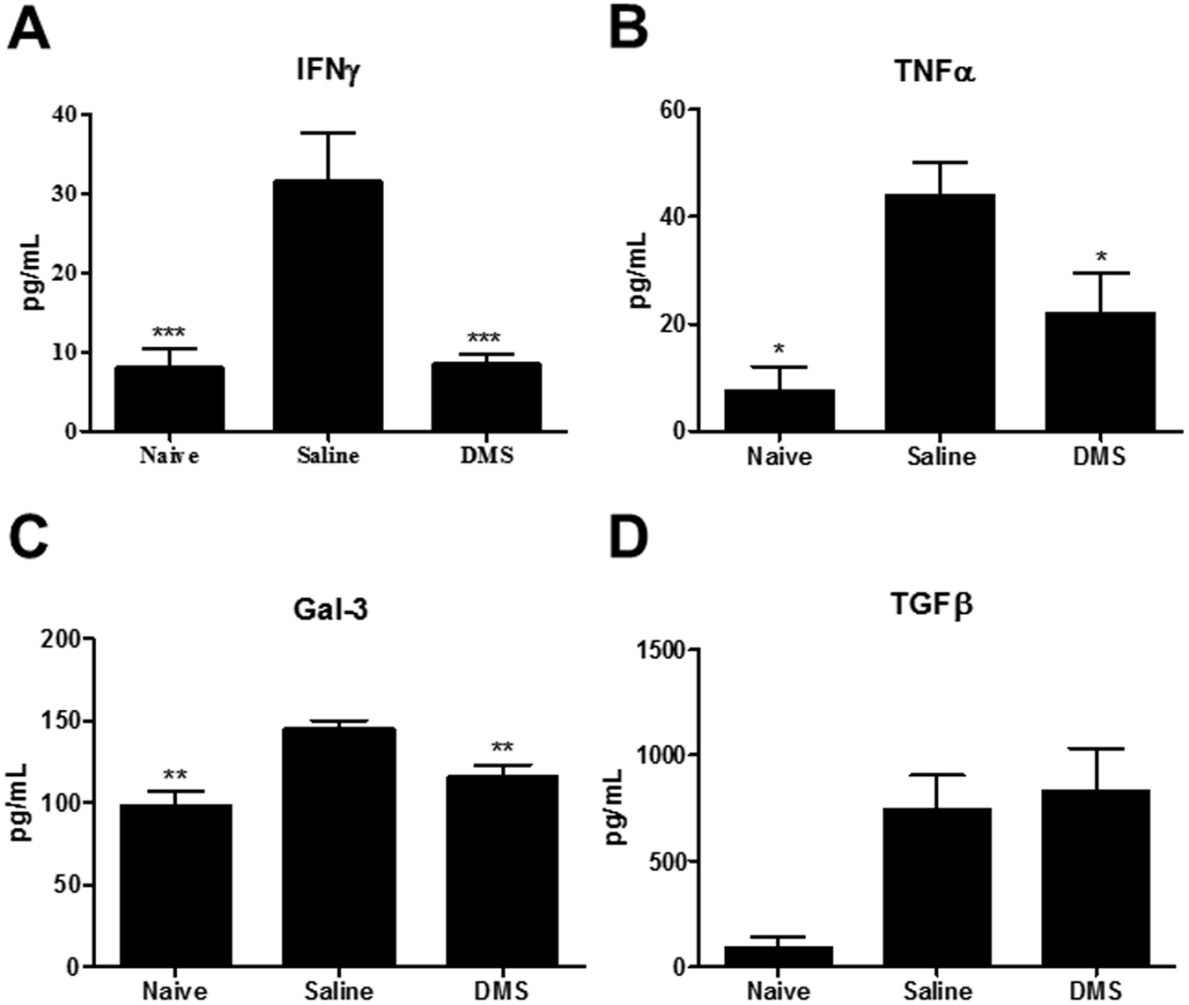
Modulation of systemic cytokine production in chronic chagasic mice treated with DMS. Concentrations of IFNγ (**A**), TNFα (**B**), Gal-3 (**C**) and TGFβ (**D**) in the sera from naive and chagasic mice treated with saline or DMS. Values represent means ± SEM of 10 mice/group. ***P < 0.001; **P < 0.01; *P < 0.05 compared to saline group.

**Figure 3:**
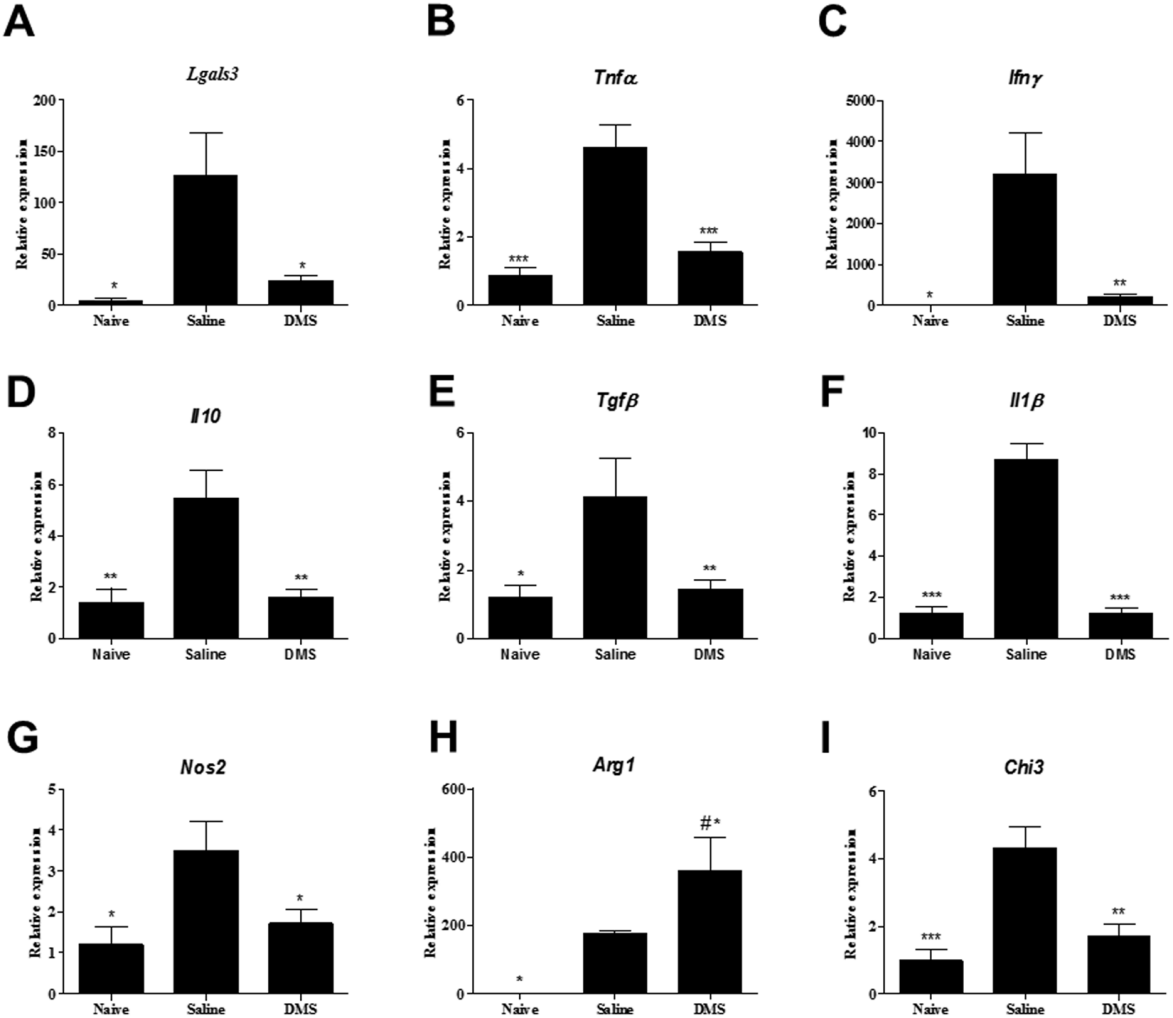
Gene expression in the hearts of infected mice after DMS treatment. Analysis of gene expression was performed by real-time qRT-PCR using cDNA samples prepared from mRNA extracted from hearts of naive and chronic Chagasic mice treated with saline or DMS. (**A**) Gal-3, (**B**) TNFα, (**C**) IFNγ, (**D**) IL-10, (**E**) TGFβ, (**F**) IL-1β, (**G**) NOS2, (**H**) ARG1 and (**I**) CHI3 gene expression. Bars represent means ± SEM of 10 mice/group. ***P < 0.001; **P < 0.01; *P < 0.05 compared to saline group; ^#^ *P* < 0.05 compared to naive group.

We have previously shown the overexpression of galectin-3 in the hearts of chronic chagasic mice^13^. To evaluate the effects of DMS on the expression of this important mediator of inflammation and fibrosis, we performed confocal microscopy analysis in the heart tissue. Vehicle-treated, *T. cruzi*-infected mice had a high expression of galectin-3, while a reduction of galectin-3 expression was seen after DMS treatment (Fig. 1B). Morphometrical analyses revealed a statistically significant difference between the groups (Fig. 1F). Moreover, DMS treatment also caused a significant reduction in the concentration of galectin-3 in the serum of *T. cruzi*-infected mice (Fig. 2C), as well as in the expression of galectin-3 gene in the hearts (Fig. 3A).

### DMS administration modulates the production of inflammatory mediators in *T. cruzi*-infected mice

CCC has been associated with an increase of IFNγ and TNFα production in mice, as well as in humans^14, 15^. We observed, both in the sera as well as in the heart, an up regulation in the expression of these two proinflammatory cytokines in the saline-treated chagasic mice, compared to uninfected mice (Figs 2 and 3). The administration of DMS promoted a significant reduction in the concentrations of both cytokines in the sera (Fig. 2A,B), as well as in the expression of their genes in the heart tissue (Fig. 3B,C). We also investigated the production of regulatory cytokines IL-10 and TGFβ, which are increased in *T. cruzi*-infected mice. TGFβ concentrations in the sera were similar in both vehicle and DMS-treated infected groups, and increased compared to naive mice (Fig. 2D). The expression of IL-10 gene in the hearts, also increased by *T. cruzi* infection, was reduced after DMS treatment compared to saline group (Fig. 3D). Since macrophages are one of the main cell populations composing the heart inflammatory infiltrate in Chagas disease^15^, we investigated the expression of genes associated with macrophage activation. IL-1β expression in the heart was found to be increased by *T. cruzi* infection and significantly reduced by DMS treatment (Fig. 3F). The expression of iNOS, a marker of M1 activation increased in the hearts of *T. cruzi*-infected mice, was reduced after DMS treatment (Fig. 3G). When M2 activation markers were analyzed, we observed an up regulation of Arg1 gene expression after DMS, while CHI3 was down-regulated in the hearts of DMS-treated mice, when compared to saline-treated controls (Fig. 3H,I).

### DMS improves exercise capacity, reduces parasitism but does not ameliorate cardiac electric disturbances

**Figure 4:**
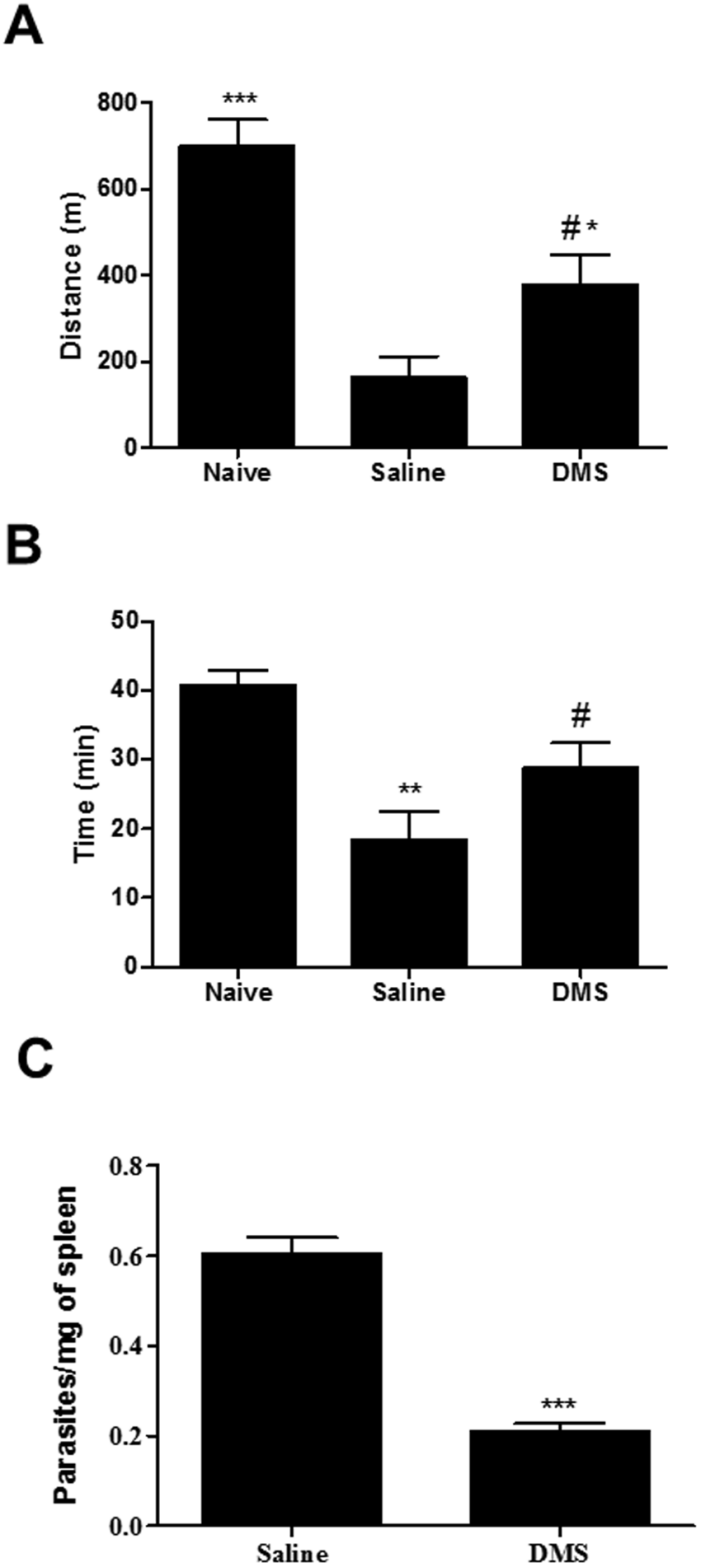
Effects of DMS treatment in cardiac function and parasite load. After an adaptation period in the treadmill chamber, naive and saline-treated or DMS-treated chronic Chagasic mice exercised at 5 different velocities (7.2, 14.4, 21.6, 28.8 and 36.0 m/min), with increasing velocity after 5 min of exercise at a given speed. (**A**) Distance run and (**B**) Time of exercise on a motorized treadmill. (**C**) Spleen fragments obtained from normal and *T. cruzi*-infected mice treated with saline or DMS were used for DNA extraction and qRT-PCR analysis for quantification of parasite load (primer 1 5′-GTTCACACACTGGACACCAA-3′ and primer 2 5′-TCGAAAACGATCAGCCGAST-3′). The standard curve of DNA ranged from 4.7 × 10^−1^ to 4.7 × 10^6^). Bars represent means ± SEM of 8–10 mice/group. ***P < 0.001; **P < 0.01; *P < 0.05 compared to saline group; ^#^ *P* < 0.05 compared to naive group.

The exercise capacity of the experimental groups was evaluated before and after treatment. *T. cruzi*-infected mice ran less time and smaller distance when compared to naive controls (Fig. 4A,B). DMS-treated mice, however, showed a better performance in the treadmill test when compared to saline-treated controls. The majority of *T. cruzi*-infected mice presented severe cardiac conduction disturbances in the EKG records, such as AV blockage, intraventricular conduction disturbances and abnormal cardiac rhythm, six months after infection. At the end of treatment, no improvements were observed in DMS-treated mice, and all *T. cruzi*-infected mice aggravated the conduction disturbances during the observed time (Table 1). To investigate whether the anti-inflammatory response induced by DMS treatment affected the immune response against the parasite, we analyzed the residual *T. cruzi* infection by qRT-PCR in the spleens of infected mice. As shown in Fig. 4C, a significant reduction of parasite load was observed in DMS-treated mice compared to saline-treated controls.

**Table 1:** Number of animals per arrhythmias before treatment (Pre) and at the end of treatment (Post) in naive (CTRL), chronic infected and vehicle-treated (INF + Saline) and chronic infected, DMS-treated (INF + DMS) groups.

	NSR		1^st^ AVB		IACD		JR		IVCD		VB		TAVB	
	Pre	Post	Pre	Post	Pre	Post	Pre	Post	Pre	Post	Pre	Post	Pre	Post
CTRL (n = 05)	05	05	—	—	—	—	—	—	—	—	—	—	—	—
INF + Saline (n = 11)	—	—	07	02	—	01	01	—	03	—	01	—	03	06
INF + DMS (n = 11)	03	—	02	01	—	01	02	02	03	—	03	02	02	04

In infected groups, some animals developed more than one type of arrhythmias. NSR = Normal sinus rhythm, 1^st^ AVB = 1^st^ degree atrio-ventricular block, IACD = Intra-atrial conduction disturbance, JR = Junctional rhythm, IVCD = Intra-ventricular conduction disturbance, VB = Ventricular bigeminism, TAVB = Total atrio-ventricular dissociation.

### Modulation of lymphocyte and macrophage functions *in vitro* by DMS

The inflammatory infiltrate in the hearts of *T. cruzi*-infected mice is mainly composed by T lymphocytes and macrophages^15^. Thus, we tested the effects of DMS *in vitro* in these two cell populations. To investigate whether DMS can directly modulate the activation of lymphocytes, we assessed the proliferation of splenocytes stimulated by concanavalin A (Con A) or anti-CD3 plus anti-CD28. A concentration-dependent inhibition of lymphoproliferation was seen when DMS was added to the cultures (See Supplementary Fig. S1A and B). Additionally, the production of IL-2 and IFNγ upon Con A stimulation was significantly reduced by DMS (See Supplementary Fig. S1C and D). Dexamethasone, a known immunosuppressive agent, reduced proliferation and cytokine production (See Supplementary Fig. S1). Moreover, the addition of DMS to macrophage cultures activated by LPS plus IFNγ caused an increase in IL-1β production (See Supplementary Fig. S2A). In contrast, the production of other inflammatory mediators, such as TNFα, IL-6, IL-10 and nitric oxide was reduced in a concentration-dependent manner (See Supplementary Fig. S2B–E). NF-κB activation participates in the regulation of several pro-inflammatory genes, including TNFα. To investigate whether DMS acted through the modulation of NF-kB activation, we performed an assay using RAW cells transduced with a reporter gene under the control of a promoter regulated by NF-κB. As shown in Supplementary Fig. S2F, DMS at 10 and 5 µM caused about 20% reduction of luciferase activity induced by activation with LPS and IFNγ. To understand if DMS effects in macrophages has off-targets effects by the inhibition of PKC and MAPK, we tested the action of DMS in the presence of specific inhibitors of ERK-1/2 (PD98059) and MAPK (BIS). The inhibition of IL-6 and iNOS production by DMS was not affected by the inhibitors (See Supplementary Fig. S2G and H).

### Antiparasitic effects of DMS *in vitro*

**Figure 5:**
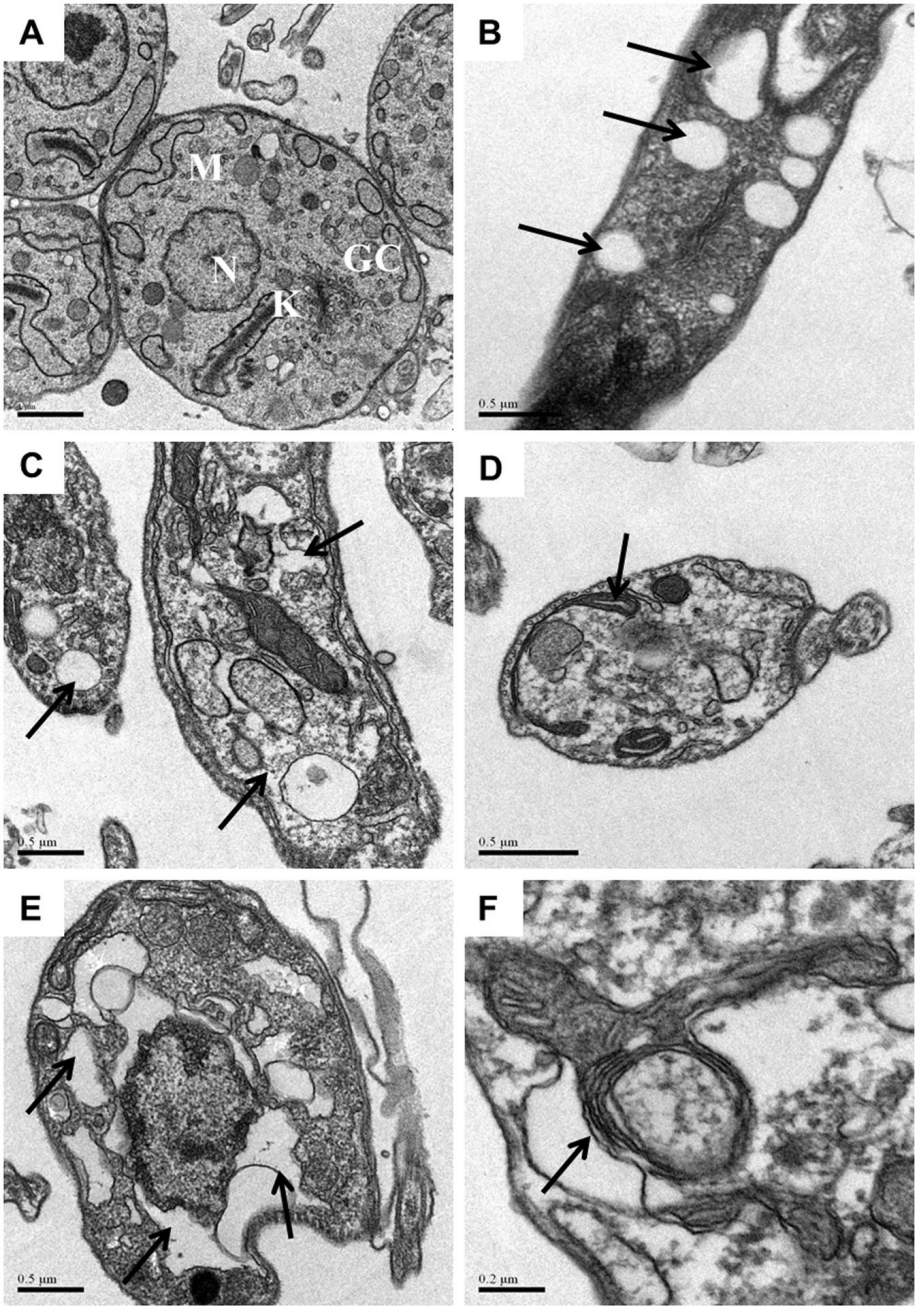
Transmission electron micrographs of trypomastigotes treated or not with DMS for 24 h. (**A**) Untreated trypomastigotes presenting a typical morphology of the nucleus (N), kinetoplast (K), mitochondria (M) and Golgi complex (GC). (**B**,**C**) Trypomastigotes treated with DMS (1 µM) causes the formation of numerous and atypical vacuoles within the cytoplasm accompanied by a large loss of density. (**D**,**E**) Trypomastigotes treated with DMS (2 µM) shows degeneration of mitochondria and intense vacuolization. (**F)** Trypomastigotes treated with DMS (4 µM) shows myelin-figures. Black arrows indicate alterations cited. Scale bars: A = 1 µm; B–E = 0.5 µM; F = 0.2 µm.

**Figure 6:**
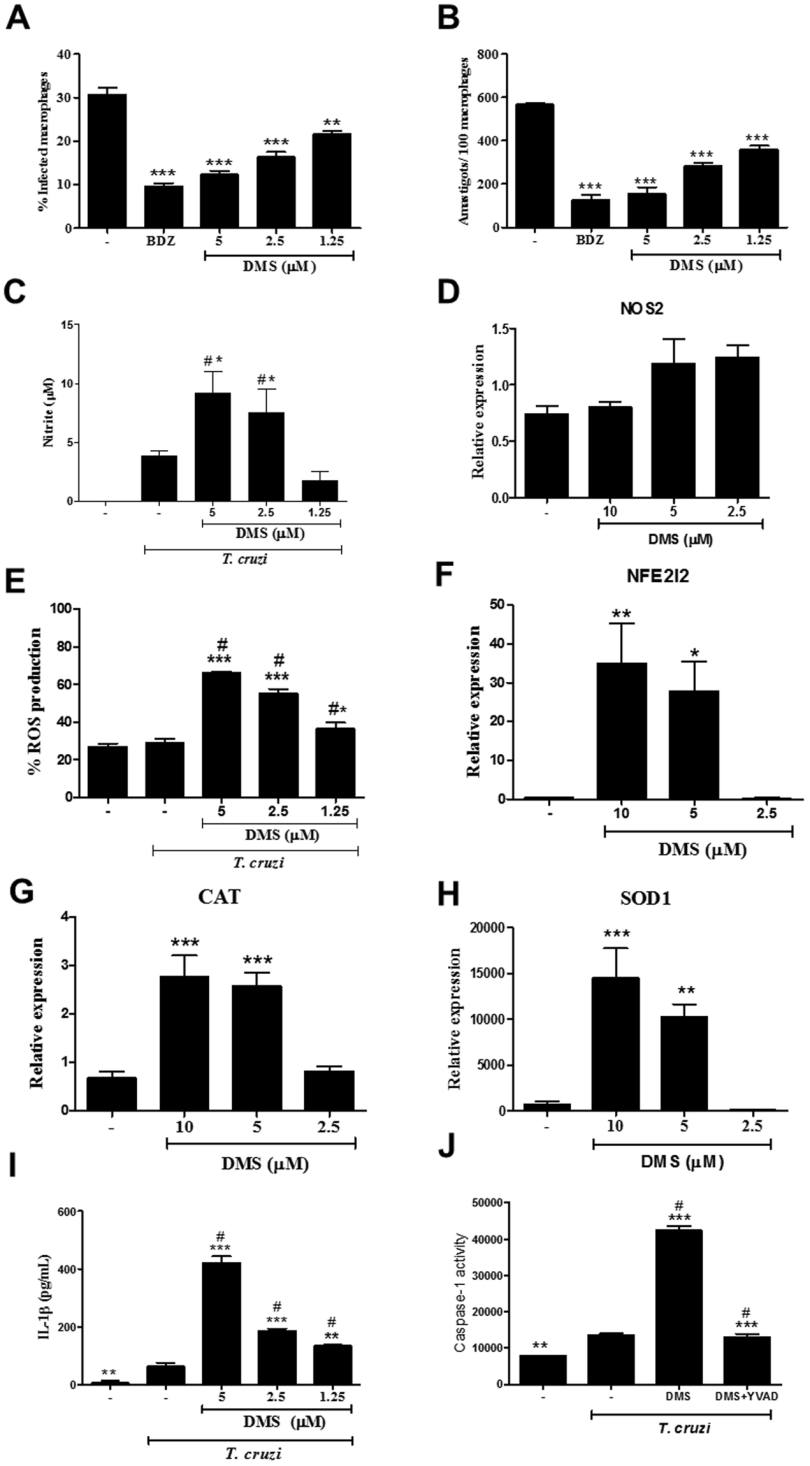
DMS inhibits amastigote proliferation in *T. cruzi*-infected macrophages, increases NO and ROS and activates caspase 1. The percentage of infected macrophages (**A**) and the relative number of amastigotes per 100 macrophages (**B**) were determined by counting hematoxylin and eosin-stained cultures after 72 hours of treatment. (**C**) Nitric oxide was determined by Griess method after 72 hours of treatment. (**D**) Relative expression of NOS2 gene in infected macrophages treated or not with DMS. (**E**) Reactive oxygen species was quantified by stained with 2′,7′-dichlorofluorescin diacetate after 30 minutes of treatment. (**F**–**H**) Relative expression of NFE2I2, CAT and SOD1 genes in infected macrophages treated or not with DMS. (**I**) IL-1β production quantified by ELISA. (**J**) Caspase-1 activity measured using caspase-Glo 1 inflammasome assay in cultures incubated with complete medium alone, with DMS (5 μM) or with DMS and YVAD (a caspase-1 inhibitor) in triplicate for 2 h. Values represent means ± SEM of 4 determinations. ***P < 0.001; **P < 0.01; *P < 0.05 compared to infected and untreated group; ^#^ *P* < 0.05 compared to uninfected and untreated group.

To investigate the mechanisms by which DMS caused the reduction on parasite load *in vivo*, we evaluated the antiparasitic activity of DMS *in vitro*. To determine whether DMS acts directly on the parasite, we analyzed the effects of DMS on *T. cruzi* trypomastigote cultures (Table 2). Addition of DMS at various concentrations in axenic cultures of *T. cruzi* trypomastigotes allowed the determination of the EC_50_ value at 1.98 µM, while benznidazole presented an EC_50_ of 12.53. Regarding the cytotoxicity of DMS, we determined the CC_50_ in mouse macrophage cultures at 9.02 µM. Next, we investigated the mechanisms of cell death in trypomastigotes forms of *T. cruzi* induced by DMS, by flow cytometry analysis. Incubation with DMS induced apoptosis of trypomastigotes in a concentration-dependent manner, as shown by the increase in annexin positive cells (see Supplementary Fig. S3). Furthermore, we evaluated the morphology and ultrastructure of trypomastigotes incubated with DMS. Compared with untreated trypomastigotes, parasites exposed to DMS for 24 h exhibited the formation of numerous and atypical vacuoles within the cytoplasm, a large loss of density degeneration of mitochondria and intense vacuolization (Fig. 5A–E). Interestingly, we also observed the presence of myelin-like figures within the cytoplasm (Fig. 5F), which is suggestive of parasite starvation or autophagy induced by DMS. The antiparasitic effects of DMS on the intracellular form of the parasite were also investigated. Macrophages infected *in vitro* by *T. cruzi* had a concentration-dependent reduction in the number of amastigotes and in percentage of infection (Fig. 6A,B). DMS at 5 µM presented a similar effect when compared to benznidazole, a standard anti-*T. cruzi* chemotherapy agent (Fig. 6A,B). *T. cruzi*-infected macrophage cultures had an increased production of nitric oxide (Fig. 6C). Addition of DMS caused a concentration-dependent increase of nitric oxide by infected macrophages (Fig. 6C). The transcription of inducible nitric oxide synthase (iNOS) gene, however, was not altered by DMS treatment (Fig. 6D). We also evaluated the production of reactive oxygen species (ROS) in *T. cruzi*-infected macrophage cultures. DMS induced a concentration-dependent increase of ROS (Fig. 6E), as well as the transcription factor NFE2l2, which regulates the expression of key protective enzymes against ROS (Fig. 6F). Additionally, the gene expression of catalase and superoxide dismutase 1, two enzymes involved in ROS degradation, was increased by DMS treatment (Fig. 6G,H).

**Table 2:** Host cell cytotoxicity and trypanocidal activity of DMS on trypomastigotes forms of *T. cruzi* (Colombian strain).

Compound	CC_50_ MØ (µM)^a^	EC_50_ Try. (µM)^b^	SI
**DMS**	9.02 (±0.12)	1.98 (±0.47)	4.5
**BDZ**	>50	12.53 (±0.55)	>4
**GV**	0.48 (±0.05)	—	—

^a^Cell viability of mouse macrophages determined 72 h after treatment. ^b^Trypanocidal activity determined 24 h after incubation with compounds. ^b^Values represent the mean ± SEM of triplicate. Three independent experiments were performed. EC_50_ = effective concentration at 50%. CC_50_ = cytotoxic concentration at 50%. BDZ = benznidazole. GV = Gentian violet.

### Activation of inflammasome pathways in *T. cruzi*-infected macrophages

**Figure 7:**
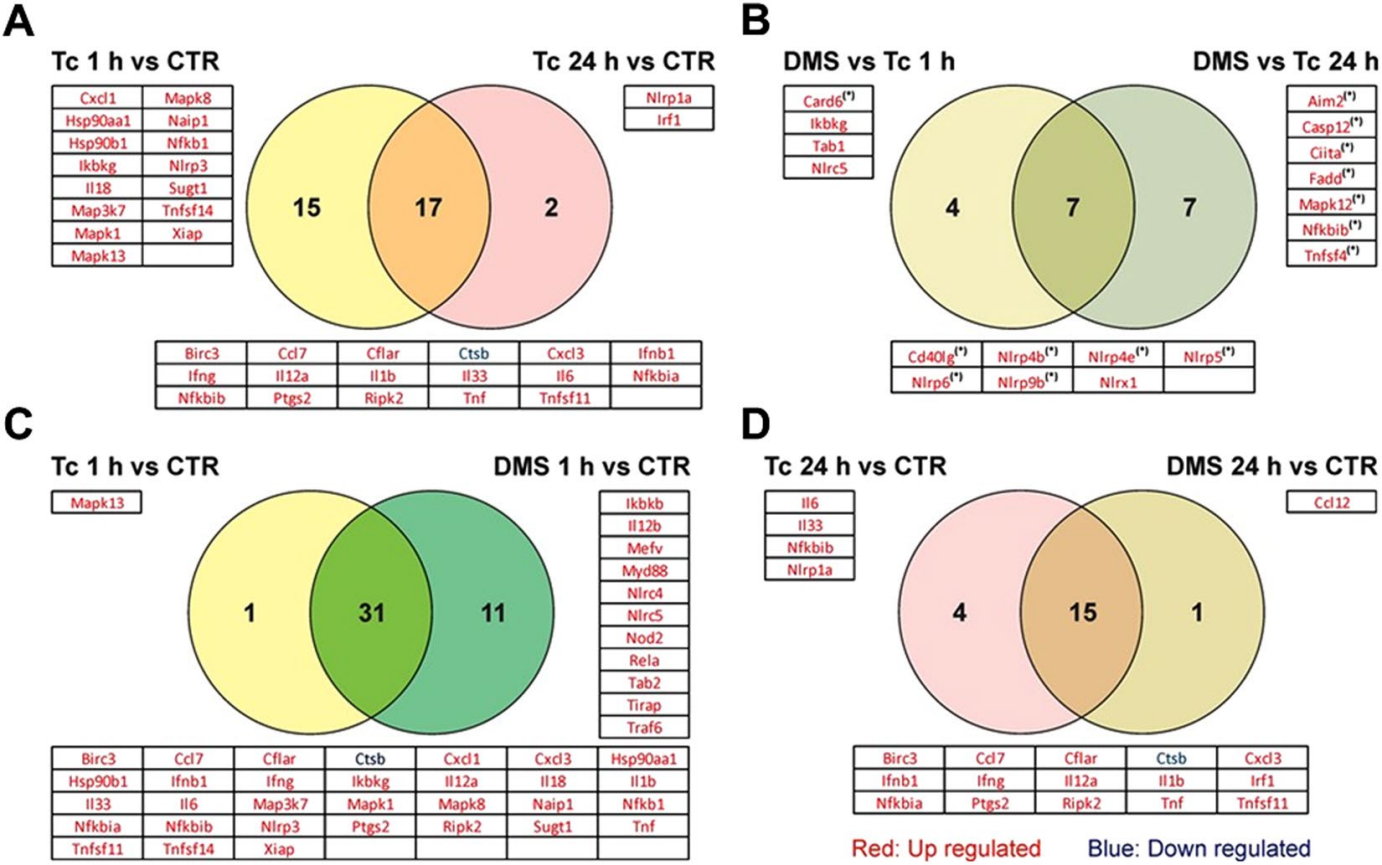
Venn diagrams representing differentially expressed genes (DEG) through *T. cruzi* infection with or without DMS treatment. (**A**) Comparison between DEG after 25 h (Tc 1 h condition) or 48 h (Tc 24 h condition) after *T. cruzi* infection with respect to uninfected macrophages (CTR condition). (**B**) Analysis of common DEG by DMS treatment for 1 h (DMS 1 h) and 24 h (DMS 24 h) when compared to infected macrophages. (**C**) Comparison between DEG by *T. cruzi* infection with or without 1 h DMS treatment with respect to uninfected macrophages. (**D**) Comparison between DEG during 48 h infection with or without 24 h DMS treatment with respect to uninfected macrophages. (*) indicates genes with |FC| ≥ 2 but p-values > 0.05.

**Figure 8:**
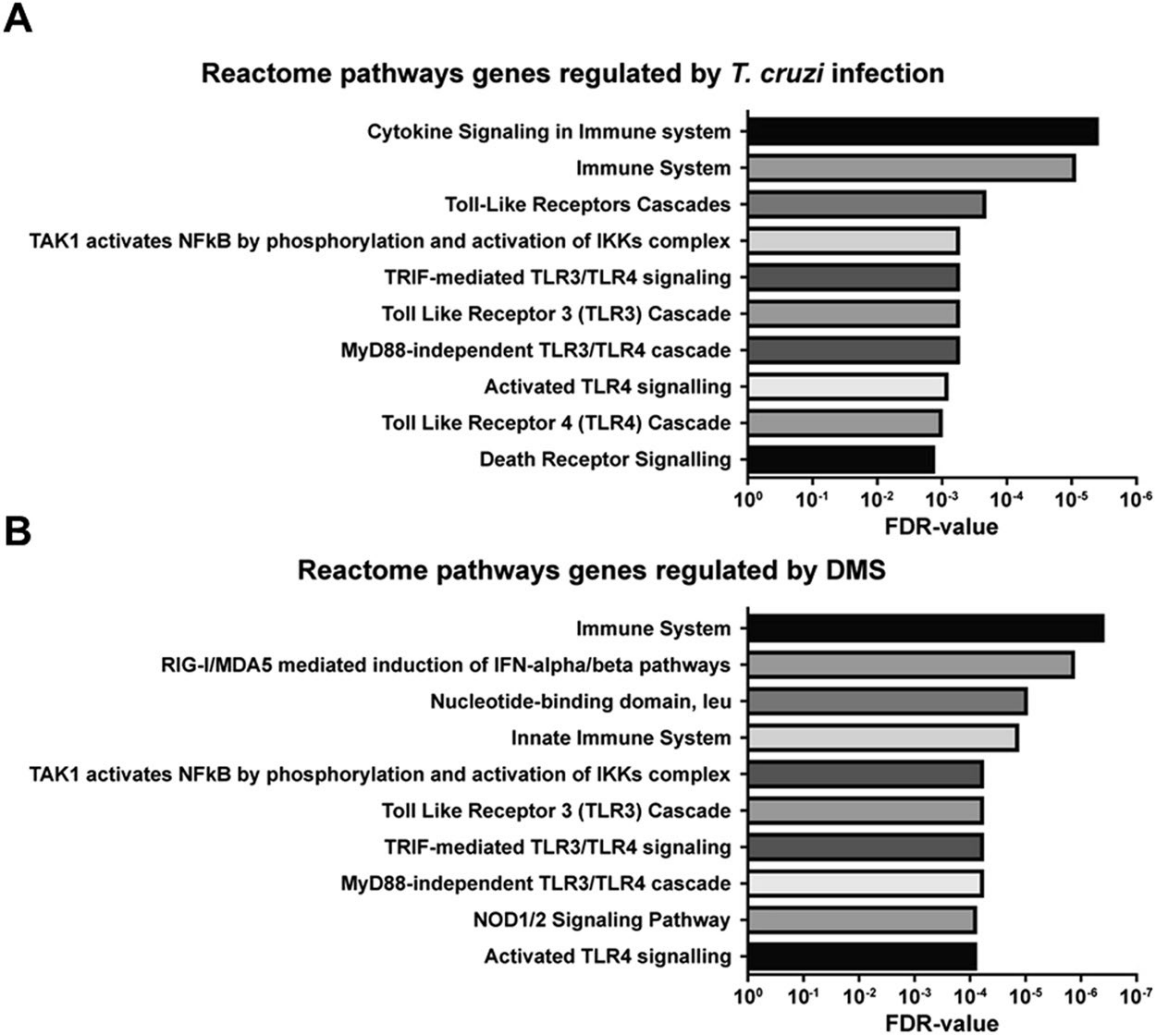
Reactome pathway analysis of DEG by *T cruzi* infection or DMS treatment. (**A**) Graph representing the 10 most significant pathways in which common DEG by *T. cruzi* at 25 h (Tc 1 h condition) and 48 h (Tc 24 h condition) after infection are involved. (**B**) Graph representing the 10 most significant pathways in which genes induced by DMS treatment are involved.

*T. cruzi*-infected macrophages incubated with DMS increased IL-1β production, in a concentration-dependent manner (Fig. 6I), suggesting involvement of an inflammasome pathway activation. To confirm that DMS induced inflammasome activation in *T. cruzi*-infected macrophages, we performed a caspase 1 activity assay. Addition of DMS (5 µM) to *T. cruzi*-infected macrophages significantly increased the activation of caspase 1, whereas infection by *T. cruzi* alone induced a slight increase of caspase 1 activity (Fig. 6J). The effects of DMS on caspase 1 activation were abrogated by the addition of the caspase 1 inhibitor YVAD (Fig. 6J). To evaluate the regulation of inflammasome pathways by DMS, we performed a qRT-PCR array for inflammasome, including genes involved in innate immunity and NOD-like receptor (NLR) signaling. Macrophages infected with *T. cruzi* for 24 h were incubated with DMS (5 µM) during 1 and 24 h and total RNA was extracted for gene expression analysis, compared to uninfected macrophage cultures (see Supplementary Tables 1–7). *T. cruzi* infection alone activated the transcription of genes related to inflammasome pathways, including *Nlrp3*, as well as several genes coding for chemokines and cytokines. Additionally, several genes related to signaling transduction pathways, including Mapk and NF-κB pathway were upregulated (Fig. 7A). Analysis using the reactome pathway database highlighted the main pathways activated by *T. cruzi* infection, which include immune system related genes, NF-κB and TLR pathways (Fig. 8A). The transcription of 17 genes were regulated by *T. cruzi* infection at the two time points evaluated. When changes in *T. cruzi*-infected macrophages at the two time points were compared, we found the expression of 15 genes altered only at 1 h time point (25 h of infection), whereas 2 genes were found altered only at 24 h time point (48 h of infection) (Fig. 7A). DMS treatment alone (24 h) did not induce any gene transcription change (see Supplementary Table S1). When *T. cruzi*-infected macrophages treated or not with DMS were compared, however, we found that DMS treatment suppressed the *T. cruzi* upregulated expression of *Mapk13* (Fig. 7C), *Il6*, *Il33*, *Nfkbib* and *Nlrp1a* (Fig. 7D). Additionally, DMS treatment increased the activation of *Nlrc5* and *Nlrx1* genes, and had an increasing trend of expression of *Nlrp4*, *Nlrp5*, *Nlrp6*, *Nlrp9* genes when compared to *T. cruzi*-infected cells (Fig. 7B). Analysis using the reactome pathway database showed that, in addition to the pathways induced by *T. cruzi* infection, DMS treatment favoured the activation of RIG and NOD signaling pathways (Fig. 8B).

## Discussion

Persistent inflammation is one of the hallmarks of chronic Chagas disease cardiomyopathy, and leads to a progressive destruction of the myocardium and heart dysfunction^5, 6^. Therefore, the development of therapeutic strategies aiming at modulation of inflammation without affecting parasite control is of great interest. Here we show, using a mouse model of chronic Chagas cardiomyopathy which reproduces the pathological findings observed in human hearts, a potent effect of DMS, causing reduction of heart inflammation and fibrosis, modulation of pro-inflammatory mediators and improvement of exercise capacity. Importantly, the residual parasite load found in mice chronically infected with *T. cruzi* was reduced by DMS treatment.

In our study, a marked reduction of inflammation was seen after DMS treatment, as shown by two different analyses, morphometry and qRT-PCR for quantification of CD45 expression. Moreover, the production of IFNγ and TNFα, two cytokines known to promote the chronic Chagas myocarditis, were significantly reduced in the heart, as well as in the serum, showing a local and a systemic effect of DMS. The fact that DMS modulates the activation of lymphocytes and macrophages *in vitro* reinforces that, in addition to a reduction of cell migration, a direct effect of the drug on immune cells may cause the potent immunomodulatory effect of DMS observed *in vivo* in our model. In line with this idea, it was recently shown that FTY720, a recently approved drug that inhibits the S1P pathway, also modulates the activation of human T lymphocytes and leads to a reduction of IFNγ production^16^.

All of the three major S1P receptor (1–3) subtypes are also expressed in cardiac fibroblasts and participate in cardiac remodeling by the activation of signaling pathways through S1P. Moreover, both FTY720 and DMS have been shown to reduce fibrosis^17–19^. The expression of SphK1 is an important factor regulating the proliferation of cardiac fibroblasts^20^. SphK1-transgenic mice which overproduces endogenous S1P showed 100% occurrence of cardiac fibrosis, involved with activation of the S1P3-Rho family small G protein signaling pathway and increased ROS production^21^. Additionally, DMS-treated mice had a reduced expression of TGFβ and galectin-3, two pro-fibrogenic factors that stimulate the proliferation and production of extracellular matrix proteins by cardiac fibroblasts^22, 23^. Here we observed a reduction on fibrotic area in the heart of DMS-treated mice, corroborating the importance of S1P signaling for heart fibrosis.

An important finding was the antiparasitic effect of DMS, leading to a reduction of parasite load *in vivo*, despite causing a reduction on the production of pro-inflammatory factors, such as IFNγ and TNFα, known to play important roles in resistance to *T. cruzi* infection^24, 25^. We showed here that DMS has not only a direct effect on the parasite, causing several cellular alterations and death of trypomastigote forms, but also an indirect effect, by inducing the increase of NO and ROS production in infected macrophages *in vitro*. These results are in accordance with a previous report showing S1P down regulating iNOS expression in macrophages through the inhibition of NF-κB, AP-1 and/or STAT-1 activation^26^. This suggests that the inhibition of S1P production by DMS treatment may lead to an increased NO production, also contributing to the *in vivo* antiparasitic mechanisms of DMS.

Chagas disease cardiomyopathy may result from multiple pathological mechanisms, including immune responses against the parasite, as well as self-reactive responses against cardiac antigens^27^. The reduction of parasitism by benznidazole, a drug used to treat Chagas disease, reduces cardiac alterations during the chronic phase of infection^28^. Additionally, the reinforcement of immunological tolerance to myocardial antigens also caused reduction of inflammation in a mouse model of *T. cruzi* infection^29^. Thus, the fact that DMS affects both inflammatory cells as well as the parasite suggests that these effects may contribute to the modulation of inflammation seen after DMS treatment.

SphK is a highly conserved enzyme in eukaryotes, and while there are two isoforms in mammals, only one is found in trypanosomatids. Depletion of *Trypanosoma brucei* SphK causes attenuation of cell division, microtubule elongation at the posterior tip, and altered organelle positioning. SphK inhibitors, such as DMS and safingol, are toxic to *T. brucei*, both of them having a 10-fold therapeutic index versus human cells, suggesting their potential use against *T. brucei* infection^30^. Here we demonstrated that DMS also induces morphological alterations and death in *T. cruzi*, suggesting that TcSphK may be a candidate target for drug development against *T. cruzi*. The reduced parasite load on infected macrophages may be also an important mechanism to reduce the dissemination of parasites, since it is likely that *T. cruzi*-infected macrophages circulate *in vivo* ^31^.

A previous study has shown that Sph and its analogues DMS and FTY720 act as a danger-associated molecular patterns (DAMPs), inducing mature IL-1β secretion and promoting inflammasome activation^32^. Both Sph analogues are capable of inducing IL-1β production, and our data reinforces the ability of DMS to activate the inflammasome pathway. *T. cruzi* infection has also been shown to induce inflammasome activation in mice, being a resistance factor to infection by regulating the production of nitric oxide and ROS^33–35^. Here we found that DMS induced inflammasome activation in *T. cruzi*-infected macrophages, as shown by increased caspase-1 activation and IL-1β production. Moreover, DMS increased the production of ROS and NO, which may be contributing to parasite clearance by infected macrophages. Interestingly, the increase on production of nitric oxide was not accompanied by an upregulation of iNOS gene transcription, suggesting that post-transcriptional and post-translational mechanisms are responsible for DMS-induced NO production^36^. The transcription of genes activated in response to ROS production, however, were increased in DMS-treated macrophages, such as NFEl2l, SOD-1 and catalase 1.

In addition to its effects on Sphk, DMS was shown to inhibit the activity of protein kinase C and mitogen-activated protein kinase (MAPK)^37, 38^. Although we did not see interference of PKC and MAPK inhibitors on the effects of DMS in macrophage cultures, further studies are required to demonstrate whether the beneficial effects of DMS *in vivo* are solely dependent on Sphk inhibition. The fact that DMS may have off-target effects, however, may not be detrimental, since FTY720 and other approved drugs are known to have off-target effects^39^.

Despite the increased production of IL-1β by DMS-treated macrophages, a reduction of TNFα and IL-6, two proinflammatory cytokines, was found. This may be due to a modulation of NF-κB activation by DMS, as observed in our study and in previous reports^40, 41^. The analysis by PCR array performed in our study indicates that DMS induces the expression of inflammasome genes known to repress NF-κB activation, such as *Nlrc5* and *Nlrx1* ^42, 43^, which suggests that inhibition of *T. cruzi*-induced NF-kB may be linked to the activation of these inflammasome mediators. Both Sph and its analogue FTY720 have already been described as regulators of the NLRP3-inflammasome and IL-1β release from macrophages^32^. Although NLRP3-inflammasome activation plays a significant role in the activation of IL-1β/ROS and NF-kB signaling of cytokine gene expression for *T. cruzi* control in human and mouse macrophages, it was observed that NLRP3-mediated IL-1β/NF-kB activation is dispensable since it is compensated by ROS-mediated control of *T. cruzi* replication and survival in macrophages^35^. Therefore, our results suggest that, in addition to the NLRP3 inflammasome previously described^32^, DMS activates other inflammasome pathways.

In conclusion, our results demonstrate a potent effect of DMS *in vitro* and *in vivo* by its antiparasitic and immunomodulatory effects and suggest that inflammasome activation is a promising strategy for the development of anti-Chagas disease treatment. Further studies are required to demonstrate the usefulness of inhibitors of S1P pathway, which are being already used in the clinical setting, as potential candidates for the treatment of Chagas disease cardiomyopathy.

## Methods

### Animals

Four weeks-old male C57BL/6 mice were used in all experiments. They were raised, maintained in the animal facility of the Center for Biotechnology and Cell Therapy, Hospital São Rafael (Salvador, Bahia, Brazil), and provided with rodent diet and water *ad libitum*. All experiments were carried out in accordance with the recommendations of Ethical Issues Guidelines, and were approved by the local ethics committee for animal use under number 001/15 (FIOCRUZ, Bahia, Brazil).

### *Trypanosoma cruzi* infection and DMS treatment

Trypomastigotes of the myotropic Colombian *T. cruzi* strain were obtained from culture supernatants of infected LLC-MK2 cells. Infection was performed by intraperitoneal (i.p.) injection of 1000 *T. cruzi* trypomastigotes in saline and parasitemia was monitored during infection. Groups of chronically infected mice were treated i.p. with N,N-dimethylsphingosine (DMS; 200 µg/kg; Cayman Chemical, Ann Arbor, MI) based on a previous report by Lai and col. (2008)^44^, 3×/week during two months (Fig. 1A). Control infected mice received vehicle (saline solution) in the same regimen. Groups of mice were euthanized one week after therapy, under anesthesia with 5% ketamine (Vetanarcol®; Konig, Avellaneda, Argentina) and 2% xylazine (Sedomin®; Konig).

### Exercise capacity and electrocardiography analysis

A motor-driven treadmill chamber for one animal (LE 8700; Panlab, Barcelona, Spain) was used to exercise the animals. The speed of the treadmill and the intensity of the shock (mA) were controlled by a potentiometer (LE 8700 treadmill control; Panlab). Total running distance and time of exercise were recorded.

Electrocardiography was performed using the Bio Amp PowerLab System (PowerLab 2/20; ADInstruments, Castle Hill, NSW, Australia), recording the bipolar lead I. All data were acquired for computer analysis using Chart 5 for Windows (PowerLab). The EKG analysis included heart rate, PR interval, P wave duration, QT interval, QTc, and arrhythmias. The QTc was calculated as the ratio of QT interval by square roots of RR interval (Bazett’s formula)^45^.

### Morphometric analysis

The hearts of all mice were removed and half of each heart was fixed in buffered 10% formalin. Sections of paraffin-embedded tissue were stained by the standard hematoxylin-eosin and Sirius red staining methods for evaluation of inflammation and fibrosis, respectively, by optical microscopy. Images were digitized using a color digital video camera (CoolSnap, Montreal, Canada) adapted to a BX41 microscope (Olympus, Tokyo, Japan). Morphometric analyses were performed using the software Image Pro Plus v.7.0 (Media Cybernetics¸ San Diego, CA), as described before^46^.

### Confocal immunofluorescence analyses

Sections of formalin-fixed paraffin embedded hearts were used for detection of galectin-3 expression by immunofluorescence as described before^46^. Sections were incubated overnight with anti-galectin-3, diluted 1:50 (Santa Cruz Biotechnology, Santa Cruz, CA) followed by incubation, for 1 h, with Alexa fluor 633 (1:200) (Molecular Probes, Carlsbad, CA) Nuclei were stained with 4,6-diamidino-2-phenylindole (Vector Laboratories, Burlingame, CA). The presence of fluorescent cells was determined by observation on a FluoView 1000 confocal microscope (Olympus).

### Quantification of parasite load

For DNA extraction, spleen fragments were submitted to DNA extraction using the NucleoSpin Tissue Kit (Machenerey-Nagel, Düren, Germany), as recommended by the manufacturer. Primers were designed based on the literature^47^, and the quantification of parasite load was performed as described previously^46^. To calculate the number of parasites per milligram of tissue, each plate contained an 8-log standard curve of DNA extracted from trypomastigotes of the Colombian *T. cruzi* strain in duplicate. Data were analyzed using 7500 software 2.0.1 (Applied Biosystems).

### Macrophage infection *in vitro*

Peritoneal exudate macrophages obtained from C57BL/6 mice, four days after thioglicollate injection, were seeded at a cell density of 2 × 10^5^ cells/mL in a 24 well-plate with rounded coverslips on the bottom in RPMI-1640 medium (Sigma-Aldrich, St. Louis, MD) supplemented with 10% fetal bovine serum (FBS; Gibco Laboratories, Gaithersburg, MD) and 50 µg/mL of gentamycin (Novafarma, Anápolis, GO, Brazil) and incubated for 24 h. Cells were then infected with trypomastigotes (1:10) for 2 h. Free trypomastigotes were removed by successive washes using saline solution. Cultures were incubated for 24 h to allow full internalization and differentiation of trypomastigotes into amastigotes. Cultures were then incubated in complete medium alone or with test inhibitors for 72 h. Cells were fixed, then stained with hematoxylin and eosin, and submitted to manual counting using an optical microscope (Olympus).

### Real time reverse transcription polymerase chain reaction (qRT-PCR)

RNA was extracted of the heart samples and macrophage cultures using TRIzol (Invitrogen, Molecular Probes, Eugene, OR). cDNA was synthetized using High Capacity cDNA Reverse Transcription Kit (Applied Biosystems). The qPCR was prepared with TaqMan® Universal PCR Master Mix (Applied Biosystems). qRT-PCR assays were performed to detect the expression levels of *Ptprc*, *Lgals3, Tnf*, *Ifnγ*, *Il10*, *Tgfβ*, *Nos2*, C*hi3l3*, *Il1β*, *Arg1*, *Nfe2l2*, *Cat* and *Sod1*. All reactions were run in triplicate on an ABI 7500 Real Time PCR System (Applied Biosystems) under standard thermal cycling conditions. A non-template control (NTC) and non-reverse transcription controls (No-RT) were also included. The samples were normalized with 18S and *Hprt*. The threshold cycle (2-ΔΔCt) method of comparative PCR was used to analyse the results^48^.

### PCR array

Peritoneal exudate macrophages (2 × 10^6^ cells/mL) were incubated in 24 well-plates with supplemented RPMI for 24 h. After washing with saline solution to discard non-adherent cells, infection was performed with trypomastigotes (1:10), for 24 h. Free trypomastigotes were removed by successive washes. Cultures were then incubated with complete medium alone or with DMS (5 µM) in triplicate for 24 h. RNA was extracted using the Rneasy Plus Mini Kit (Qiagen, Valencia, CA). Quantification of RNA and degree of purity were performed in a spectrophotometer (NanoDrop ™ 1000, Thermo Fisher Scientific, Wilmington, DE). Sample integrity was observed using a 1% agarose gel. cDNA synthesis was performed using the RT2 First Strand kit (Qiagen). For target gene expression analysis, we used RT2 Profiler PCR Arrays Mouse Inflammasome (Qiagen), the SYBR®Green system and 96-well plates. The 7500 Real Time PCR was used (Applied Biosystems). All experiments were performed in DNase/RNase free conditions. The analysis was performed by Threshold Cycle Method^48^, obtained by calculating 2-ΔΔCt. The QIAGEN’s qPCR analysis web portal date, available on http://pcrdataanalysis.sabiosciences.com/pcr/arrayanalysis.php was used to assist in the analysis, and analysis of differentially expressed genes and pathways prediction were done through free online bioinformatic sites BioVenn (www.cmbi.ru.nl/cdd/biovenn) and Enrichr (amp.pharm.mssm.edu/Enrichr).

### Lymphoproliferation assay

Spleen cell suspensions from naive C57BL/6 mice were prepared in DMEM medium (Life Technologies, GIBCO-BRL, Gaithersburg, MD) supplemented with 10% FBS and 50 μg/mL of gentamycin. Splenocytes were cultured in 96-well plates at 1 × 10^6^ cells per well, in a final volume of 200 μL, in triplicate, in the presence of 2 μg/mL concanavalin A (Con A; Sigma-Aldrich) only or with anti-CD3 and anti-CD28 (Themo Fisher Scientific), in the absence or presence of DMS at different concentrations (2.5, 1.25 or 0.62 µM). After 48 h, plates were pulsed with 1 μCi of methyl-^3^H-thymidine (Perkin Elmer, Waltham, MA) for 18 h. The plates were harvested and the ^3^H-thymidine uptake was determined using a β-plate counter (Multilabel Reader, Finland). Dexamethasone (Sigma-Aldrich) was used as positive control.

### ELISA assays and determination of nitric oxide production

Serum samples from the *in vivo* study were used for galectin-3, TNFα and IFNγ and TGFβ determination. Quantification of cytokines was performed by ELISA, using specific antibody kits (R&D Systems, Minneapolis, MN), according to manufacturer’s instructions. To estimate the amount of nitric oxide (NO) produced, macrophage culture supernatants were used for nitrite determination by the Griess reaction, as previously described^49^.

### Reactive oxygen species (ROS) production assay

Thioglicollate-elicited peritoneal exudate macrophages (1 × 10^6^) were obtained and infected with *T. cruzi*. Cultures were then incubated with complete medium alone or with DMS for 30 min. After incubation, macrophages were removed from each well using 0.01% trypsin and labeled with 10 μM of 2′,7′-dichlorofluorescin diacetate (Sigma-Aldrich) for 30 minutes at 37 °C. Cells were then washed and analyzed using a cell analyzer (LSRFortessa; BD Biosciences, San Jose, CA) with FlowJo software (Tree Star, Ashland, OR).

### Caspase 1 activity assay

Thioglicollate-elicited peritoneal exudate macrophages (1 × 10^6^) were obtained and infected with *T. cruzi* as described above. Cultures were then incubated with reagents for 2 h and after that, caspase-1 activity was measured using caspase-Glo® 1 inflammasome assay (Promega, Madison, WI), according to the manufacturer’s instructions. The luminescence of each sample was measured in a Glomax 20/20 luminometer (Promega).

### Transmission electron microscopy analysis

*T. cruzi* trypomastigotes (5x10^7^) were treated with DMS (1, 2 or 4 µM) and incubated for 24 h at 37 °C. After incubation, parasites were fixed for 1 h at room temperature with 2% formaldehyde and 2.5% glutaraldehyde (Electron Microscopy Sciences, Hatfield, PA) in sodium cacodylate buffer (0.1 M, pH 7.2) for 1 h at room temperature. After fixation, parasites were processed for transmission electron microscopy as previously described^50^. Images were obtained in a JEOL TEM-1230 transmission electron microscope.

### Statistical analyses

All continuous variables are presented as means ± SEM. Data were analyzed using 1-way ANOVA, followed by Newman-Keuls multiple-comparison test with Prism 5.0 (GraphPad Software, San Diego, CA). All differences were considered significant at values of *P* < 0.05.

## Electronic supplementary material

Supplementary material

Supplementary Table S1

Supplementary Table S2

Supplementary Table S3

Supplementary Table S4

Supplementary Table S5

Supplementary Table S6

Supplementary Table S7
