# Supplementary material for: Therapeutic effects of sphingosine kinase inhibitor N,N-dimethylsphingosine (DMS) in experimental chronic Chagas disease cardiomyopathy
Source: Sci Rep. 2017 Jul 21;7:6171. doi: 10.1038/s41598-017-06275-z (PMC5522404; doi:10.1038/s41598-017-06275-z)
Supplement: Supplementary file 1 — Supplementary material [file 41598_2017_6275_MOESM1_ESM.doc]

### Therapeutic effects of sphingosine kinase inhibitor N,N-dimethylsphingosine (DMS) in experimental chronic Chagas disease cardiomyopathy

Juliana Fraga Vasconcelos^1-3^, Cássio Santana Meira^1,2^, Daniela Nascimento Silva^2^, Carolina Kymie Vasques Nonaka^2^, Pâmela Santana Daltro^2^, Simone Garcia Macambira^2,4^, Pablo Daniel Domizi^5^, Valéria Matos Borges^1^, Ricardo Ribeiro-dos-Santos^2^, Bruno Solano de Freitas Souza^1,2^ & Milena Botelho Pereira Soares^1,2*^

^1^Centro de Pesquisas Gonçalo Moniz, Fundação Oswaldo Cruz (FIOCRUZ), Salvador, BA, 40296-710, Brazil; ^2^Centro de Biotecnologia e Terapia Celular, Hospital São Rafael, Salvador, BA, 41253-190, Brazil; ^3^Escola de Ciências da saúde, Universidade Salvador, Salvador, BA, 41720-200, Brazil; ^4^Departamento de Bioquímica e Biofísica, Instituto de Ciências da Saúde, Universidade Federal da Bahia, Salvador, BA, 40110-100, Brazil; ^5^ Centro de Ciências da Saúde, Universidade Federal do Rio de Janeiro, Rio de Janeiro, RJ, 21944-970 Brazil.

* Corresponding author: Instituto Gonçalo Moniz, FIOCRUZ, Salvador, Bahia, Brazil. E-mail: [milena@bahia.fiocruz.br](mailto:milena@bahia.fiocruz.br).

**SUPPLEMENTARY FIGURES**

**
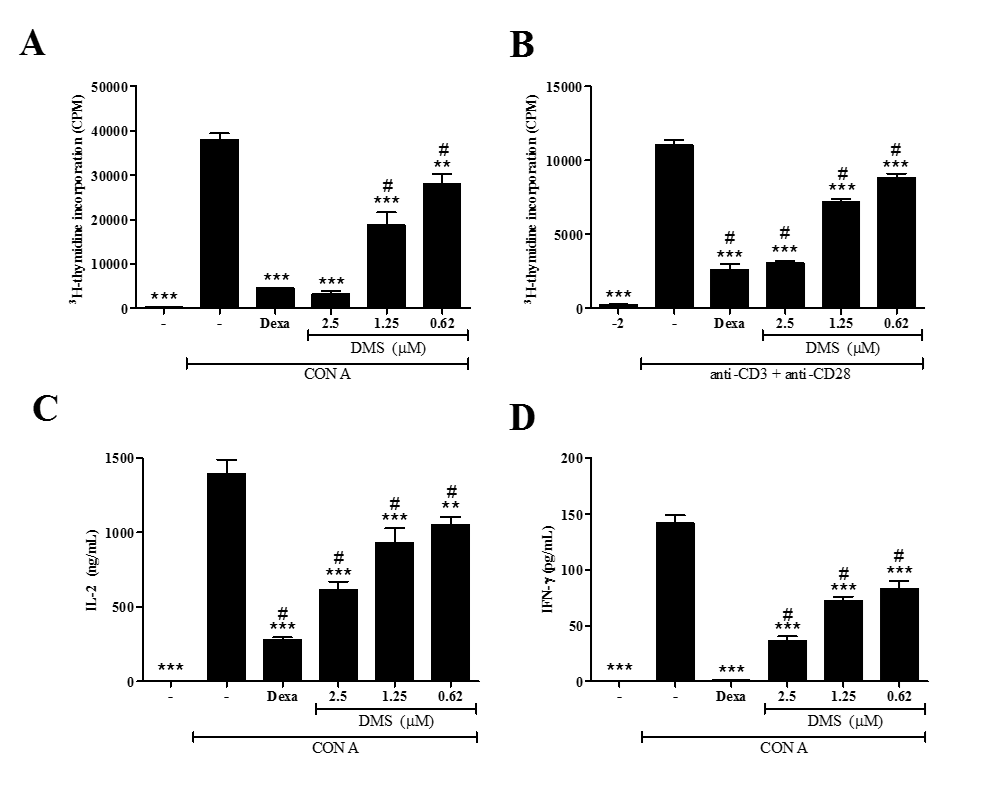
**

**Figure S1: Inhibition of lymphocyte proliferation and cytokine production by DMS.** Effect of treatments with DMS (2.5, 1.25 or 0.62 µM) or dexamethasone (2.5 µM) for 48 h on Con A **(A)** or anti-CD3 + anti-CD28 **(B)** induced lymphoproliferation respectively. Concentrations of IFNγ (**C**) and IL-2 (**D**) were determined in splenocytes treated or not with DMS or dexamethasone after 24 h of treatment. Values represent means±SEM of four determinations. ****P*<0.001; ***P*< 0.01 compared to untreated and stimulated cultures; #*P*<0.05 compared to untreated and non-stimulated cultures.

**A**

**B**

**C**

**D**

**E**

**F**

**H**

**G**

**Figure S2: Modulation of nitric oxide, cytokines and NF-κB in DMS-treated macrophages.** Effect of DMS (10, 5 or 2.5 µM) on IL-1β (**A**), IL-6 (B), TNFα (**c**), Nitrite (**D**), IL-10 **(E)** production and NF-κB activity (**F**). DMS activity on Nitrite **(G)** and IL-6 **(H)** was also investigated in the presence or absence of PD98059, an ERK-1/2 inhibitor and BIS, a PKC inhibitor. Values represent the means ± SEM of four determinations obtained in one of two experiments performed. Values represent means±SEM of 4 determinations. ****P*<0.001; ***P*< 0.01; **P*< 0.05 compared to untreated and stimulated cultures; #*P*<0.05 compared to untreated and non-stimulated cultures.

**
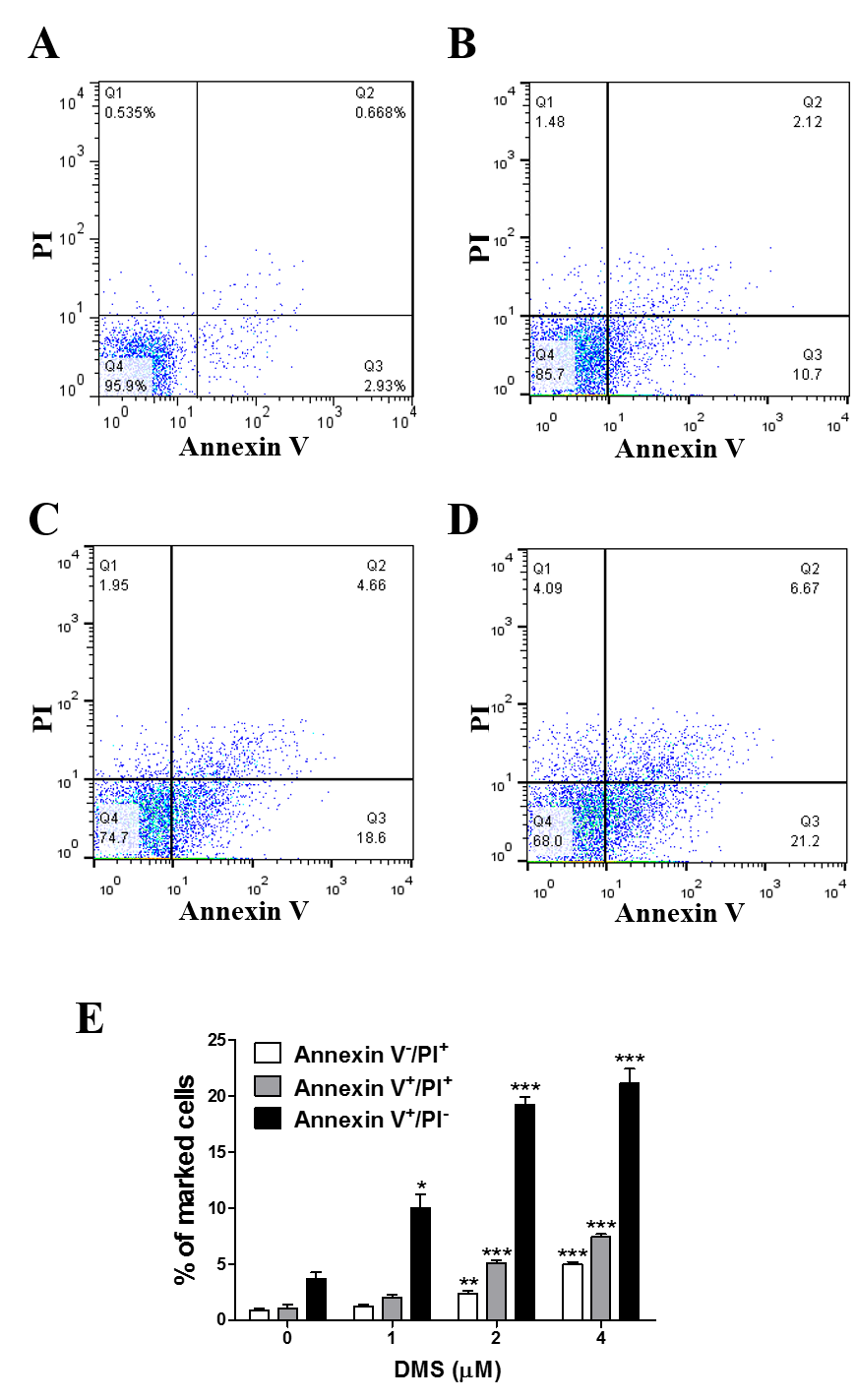
**

**Figure S3:** **DMS-based treatment causes parasite death by inducing apoptosis.** *T. cruzi* trypomastigotes were treated with DMS (1, 2 or 4 μM). Parasites were examined by flow cytometry with annexin V and PI staining. Cells plotted in each quadrant represent the following: lower left, double negative; upper left, PI single positive; lower right, annexin V single positive; upper right, PI and annexin V double positive. (**A**) Untreated parasites; (**B**) DMS at 1 μM; (**C**) DMS at 2 μM; (**D**) DMS at 4 μM. (**E**) Percentage of stained cells in each quadrant. Values are means±SEM from triplicate tests. ****P*<0.001; ***P*< 0.01; **P*<0.05 compared to untreated parasites.

**SUPPLEMENTARY METHODS**

**Lymphoproliferation assay**

Spleen cell suspensions from C57BL/6 mice were prepared in DMEM medium (Life Technologies, GIBCO-BRL, Gaithersburg, MD) supplemented with 10% fetal bovine serum (FBS; GIBCO) and 50 μg/mL of gentamycin (Novafarma, Anápolis, GO, Brazil). Splenocytes were cultured in 96-well plates at 1 × 10^6^ cells per well, in a final volume of 200 μL, in triplicate, in the presence of 2 μg/mL concanavalin A (Con A; Sigma) only or with anti-CD3 and anti-CD28 (ThemoFisher Scientific, Waltham, MA), in the absence or presence of DMS at different concentrations (2.5, 1.25 or 0.62 µM). After 48 h, plates were pulsed with 1 μCi of methyl-^3^H-thymidine (Perkin Elmer, Waltham, MA) for 18 h. The plates were harvested and the ^3^H-thymidine uptake was determined using a β-plate counter (Multilabel Reader, Turku, Finland). Dexamethasone (Sigma) was used as positive control.

**Macrophage activation**

Peritoneal exudate macrophages obtained from C57BL/6 mice, four days after tioglicolate injection, were incubated in a 24 well-plate in RPMI supplemented with 10% FBS and 50 µg/mL of gentamycin and incubated for 24 h. Macrophage cultures were activated by incubation with mouse recombinant IFNγ (5 ng/mL; Miltenyi Biotec, Bergisch Gladbach, Germany) and LPS (500 ng/mL; Sigma), in the absence or presence of DMS at various concentrations. In some experiments, cells were pretreated with 20 nM BIS (Merk-Calbiochem, Darmstadt, Germany), a PKC inhibitor or 50 μM PD98059 (Merk-Calbiochem), an ERK-1/2 inhibitor for 1 hour prior to stimulation with LPS and IFNγ and treatment with DMS for 24 h. After the incubation period, supernatants were collected for ELISA and NO determinations.

**ELISA assays and determination of nitric oxide production**

Cell culture supernatants were assayed for TNFα, IFNγ, IL-1β, IL-2, IL-6 and IL-10. Quantification of mediators was done by ELISA, using specific antibody kits (R&D System), according to manufacturer's instructions. To estimate the amount of nitric oxide (NO) produced, macrophage culture supernatants were used for nitrite determination by the Griess reaction, as previously described^1^.

**NF-**κ**B luciferase assay**

The murine mouse leukemic monocyte macrophage cell line Raw 264.7 Luc cells bearing the pBIIX-luciferase (pBIIX-luc) targeting vector containing the firefly luciferase gene (luc) driven by two NF-κB binding sites from the kappa light chain enhancer in front of a minimal fos promoter^2^ were kindly provided by Maria Célia Jamur (Department of Cell and Molecular Biology and Pathogenic Bioagentes, University of São Paulo, Ribeirão Preto, SP, Brazil). The cells were cultured in RPMI medium (Sigma) supplemented with 20% FBS and 50 μg/mL of gentamycin at 37°C in a humidified environment containing 5% CO_2_. For luciferase reporter assays, 5 × 10^5^ cells/ml were pretreated with different concentrations of DMS (10, 5 or 2.5 μM) for 1 h prior to stimulation with LPS (500 ng/mL) plus IFNγ (5 ng/mL) for 3 h. Wells were then washed with cold-PBS and cells were lysed with TNT lysis buffer (200 mM Tris, pH 8.0, 200 mM NaCl, 1% Triton X-100) for 20 min at 4°C. The determination of the luciferase activity was performed using the Promega luciferase assay system (Promega, Madison, CA), according to the manufacturer’s instructions. The samples were analyzed in a Globomax 20/20 Luminometer (Promega). Data were expressed as relative light units.

**Propidium iodide and annexin V staining**

*T. cruzi* trypomastigotes from Colombian strain (1x10^7^) were incubated for 24 h at 37 °C in the absence or presence of DMS (1, 2 or 4 µM). After incubation, the parasites were labeled for propidium iodide (PI) and annexin V using the annexin V-FITC apoptosis detection kit (Sigma-Aldrich), according to the manufacturer’s instructions. Acquisition and analyses was performed using a FACSCalibur flow cytometer (Becton Dickinson, San Diego, CA), with FlowJo software (Tree Star, Ashland, OR). A total of 10,000 events were acquired in the region corresponding to trypomastigotes forms of *T. cruzi*.

**REFERENCE**

1. Green, L.C. et al. Analysis of nitrate, nitrite, and [15N] nitrate in biological fluids. Anal. Biochem. 126, 131-138 (1982).
2. Zhong, H., SuYang, H., Erdjument-Bromage, H., Tempst, P. & Ghosh, S. The transcripitional activity of NF-kappaB is regulated by the IkappaB-associated PKAc subunit through a cyclic AMP-independent mechanism. *Cell* **89(3)**, 413-424 (1997).
