# Supplementary material for: Therapeutic effects of sphingosine kinase inhibitor N,N-dimethylsphingosine (DMS) in experimental chronic Chagas disease cardiomyopathy
Source: Sci Rep. 2017 Jul 21;7:6171. doi: 10.1038/s41598-017-06275-z (PMC5522404; doi:10.1038/s41598-017-06275-z)
Supplement: Supplementary file 2 — Supplementary Table S1 [file 41598_2017_6275_MOESM2_ESM.doc]

| **Gene symbol** | **Fold change** | | **p-value** |
| --- | --- | --- | --- |
| Aim2 | | -2.1237 | 0.204094 |
| Bcl2 | | 1.3685 | 0.879987 |
| Bcl2l1 | | 1.2407 | 0.95341 |
| Birc2 | | 1.0295 | 0.979913 |
| Birc3 | | -1.1387 | 0.377614 |
| Card6 | | -1.1825 | 0.526022 |
| Casp1 | | -1.3865 | 0.402914 |
| Casp12 | | -1.3198 | 0.702988 |
| Casp8 | | -1.441 | 0.306831 |
| Ccl12 | | -3.4123 | 0.243905 |
| Ccl5 | | -1.2612 | 0.580573 |
| Ccl7 | | -2.3679 | 0.335648 |
| Cd40lg | | -1.265 | 0.430114 |
| Cflar | | -1.238 | 0.683055 |
| Chuk | | -1.3718 | 0.188606 |
| Ciita | | -1.5557 | 0.495433 |
| Ctsb | | -1.4185 | 0.140177 |
| Cxcl1 | | -1.4998 | 0.364385 |
| Cxcl3 | | -3.114 | 0.329786 |
| Fadd | | -1.8095 | 0.238904 |
| Hsp90aa1 | | 1.0186 | 0.939451 |
| Hsp90ab1 | | -1.2481 | 0.257761 |
| Hsp90b1 | | 1.0237 | 0.916773 |
| Ifnb1 | | 1.2065 | 0.920664 |
| Ifng | | -1.5191 | 0.35276 |
| Ikbkb | | 1.4114 | 0.570729 |
| Ikbkg | | 2.0804 | 0.305958 |
| Il12a | | -1.0502 | 0.873896 |
| Il12b | | -1.4305 | 0.386283 |
| Il18 | | -1.0637 | 0.647239 |
| Il1b | | -2.9858 | 0.280212 |
| Il33 | | -1.1835 | 0.983766 |
| Il6 | | -2.4037 | 0.259644 |
| Irak1 | | 1.2479 | 0.582828 |
| Irf1 | | 1.103 | 0.88971 |
| Irf2 | | -1.062 | 0.685997 |
| Irf3 | | 1.0821 | 0.810887 |
| Map3k7 | | 1.2741 | 0.70489 |
| Tab1 | | -2.0175 | 0.649986 |
| Tab2 | | -1.0787 | 0.710648 |
| Mapk1 | | 1.1421 | 0.770892 |
| Mapk11 | | -1.0834 | 0.943307 |
| Mapk12 | | 1.6537 | 0.74755 |
| Mapk13 | | 1.0176 | 0.858 |
| Mapk3 | | 1.3772 | 0.587965 |
| Mapk8 | | -1.0706 | 0.886906 |
| Mapk9 | | 1.6518 | 0.351944 |
| Mefv | | 2.1867 | 0.751056 |
| Myd88 | | 1.7556 | 0.662173 |
| Naip1 | | -1.3077 | 0.975528 |
| Naip5 | | 1.0946 | 0.839264 |
| Nfkb1 | | 1.2433 | 0.418784 |
| Nfkbia | | -1.1058 | 0.559095 |
| Nfkbib | | 2.1244 | 0.218839 |
| Nlrc4 | | 2.4507 | 0.496826 |
| Nlrc5 | | 1.2359 | 0.882167 |
| Nlrp1a | | 1.891 | 0.86478 |
| Nlrp3 | | -1.5449 | 0.178847 |
| Nlrp4b | | -1.1415 | 0.694552 |
| Nlrp4e | | 1.3342 | 0.712576 |
| Nlrp5 | | -1.2158 | 0.66211 |
| Nlrp6 | | -1.3393 | 0.626832 |
| Nlrp9b | | -1.0637 | 0.743745 |
| Nlrx1 | | 1.7913 | 0.428779 |
| Nod2 | | 1.0099 | 0.841535 |
| P2rx7 | | 1.1909 | 0.913224 |
| Panx1 | | -1.1045 | 0.539701 |
| Pea15a | | 1.2477 | 0.783895 |
| Pstpip1 | | 1.5051 | 0.445726 |
| Ptgs2 | | -1.5656 | 0.369185 |
| Pycard | | 1.0547 | 0.862341 |
| Mok | | 1.1497 | 0.948416 |
| Rela | | 1.9968 | 0.22478 |
| Ripk2 | | -1.0768 | 0.7325 |
| Sugt1 | | 1.0166 | 0.754741 |
| Tirap | | -1.1213 | 0.55332 |
| Tnf | | -1.2472 | 0.392146 |
| Tnfsf11 | | 1.5232 | 0.791035 |
| Tnfsf14 | | -1.5825 | 0.271414 |
| Tnfsf4 | | -1.6427 | 0.495002 |
| Traf6 | | 1.6195 | 0.71653 |
| Txnip | | -1.2486 | 0.129904 |
| Xiap | | -1.03 | 0.732064 |
| Gusb | | 1.2918 | 0.375388 |
| Hprt | | -1.1862 | 0.016144 |
| Hsp90ab1 | | -1.089 | 0.661185 |
| Gapdh | | -129.6805 | 0.373901 |
| Actb | | 45.2793 | 0.493385 |

**Supplementary Table S1: Gene expression analysis between uninfected macrophages with (CTR + DMS 24 h condition) or without (CTR condition) DMS treatment during 24 h.** Fold change and p-values associated with each gene analyzed in the PCR array. Genes with higher expression (fold change value ≥ 2) in CTR + DMS 24 h condition with respect to CTR condition are highlighted in red. In blue are highlighted those genes with lower expression (fold change value ≤ -2). Changes in gene expression associated with p-value lower than 0.05 are highlighted in red.
