# Supplementary material for: Therapeutic effects of sphingosine kinase inhibitor N,N-dimethylsphingosine (DMS) in experimental chronic Chagas disease cardiomyopathy
Source: Sci Rep. 2017 Jul 21;7:6171. doi: 10.1038/s41598-017-06275-z (PMC5522404; doi:10.1038/s41598-017-06275-z)
Supplement: Supplementary file 3 — Supplementary Table S2 [file 41598_2017_6275_MOESM3_ESM.doc]

| **Gene symbol** | **Fold change** | **p-value** |
| --- | --- | --- |
| Aim2 | -1.2591 | 0.487977 |
| Bcl2 | -2.0483 | 0.343159 |
| Bcl2l1 | -5.6703 | 0.171978 |
| Birc2 | 1.5531 | 0.522543 |
| Birc3 | 6.9933 | 0.000021 |
| Card6 | -2.6593 | 0.389231 |
| Casp1 | -1.1193 | 0.587887 |
| Casp12 | -2.8026 | 0.119144 |
| Casp8 | -1.5225 | 0.279264 |
| Ccl12 | 1.4515 | 0.309795 |
| Ccl5 | 2.558 | 0.433411 |
| Ccl7 | 39.1885 | 0.000498 |
| Cd40lg | -2.3514 | 0.158427 |
| Cflar | 6.7899 | 0.006838 |
| Chuk | 1.8394 | 0.005414 |
| Ciita | -12.3827 | 0.097893 |
| Ctsb | -9.4598 | 0.006547 |
| Cxcl1 | 105.551 | 0.000003 |
| Cxcl3 | 14058.3972 | 0.000618 |
| Fadd | 1.0961 | 0.878085 |
| Hsp90aa1 | 6.5345 | 0.013761 |
| Hsp90ab1 | 1.4785 | 0.080142 |
| Hsp90b1 | 4.4274 | 0.001138 |
| Ifnb1 | 38.1001 | 0.005863 |
| Ifng | 11.2986 | 0.010946 |
| Ikbkb | 1.6332 | 0.365016 |
| Ikbkg | 14.7297 | 0.040672 |
| Il12a | 278.2188 | 0.005018 |
| Il12b | 2.4503 | 0.292899 |
| Il18 | 2.4378 | 0.002855 |
| Il1b | 450.7901 | 0.00001 |
| Il33 | 80.6856 | 0.006217 |
| Il6 | 4959.266 | 0.000561 |
| Irak1 | 1.8991 | 0.036461 |
| Irf1 | 1.3006 | 0.887258 |
| Irf2 | 1.0028 | 0.980456 |
| Irf3 | 1.0607 | 0.716242 |
| Map3k7 | 3.7689 | 0.008542 |
| Tab1 | -1.3501 | 0.437299 |
| Tab2 | 1.357 | 0.217055 |
| Mapk1 | 2.2118 | 0.041592 |
| Mapk11 | 2.789 | 0.139594 |
| Mapk12 | 2.1669 | 0.953755 |
| Mapk13 | 2.6968 | 0.018974 |
| Mapk3 | 1.6227 | 0.313136 |
| Mapk8 | 9.6492 | 0.003599 |
| Mapk9 | 1.5454 | 0.414654 |
| Mefv | 10.9799 | 0.054206 |
| Myd88 | 1.9348 | 0.444712 |
| Naip1 | 2.2899 | 0.010419 |
| Naip5 | -1.7087 | 0.268642 |
| Nfkb1 | 3.9421 | 0.008658 |
| Nfkbia | 5.3921 | 0.00045 |
| Nfkbib | 10.809 | 0.000219 |
| Nlrc4 | 9.7296 | 0.051574 |
| Nlrc5 | -1.1321 | 0.779873 |
| Nlrp1a | 3.1089 | 0.260724 |
| Nlrp3 | 11.0359 | 0.000015 |
| Nlrp4b | -2.2741 | 0.325921 |
| Nlrp4e | -2.2741 | 0.325921 |
| Nlrp5 | -2.2741 | 0.325921 |
| Nlrp6 | -2.2741 | 0.325921 |
| Nlrp9b | -2.2741 | 0.325921 |
| Nlrx1 | -2.7967 | 0.21911 |
| Nod2 | 3.1489 | 0.05662 |
| P2rx7 | 1.0387 | 0.822939 |
| Panx1 | 1.0486 | 0.852191 |
| Pea15a | -1.8168 | 0.255634 |
| Pstpip1 | -4.4903 | 0.093837 |
| Ptgs2 | 389.4455 | 0.005697 |
| Pycard | 1.2828 | 0.970053 |
| Mok | 2.4637 | 0.137541 |
| Rela | 2.2334 | 0.202473 |
| Ripk2 | 6.5651 | 0.000222 |
| Sugt1 | 3.5491 | 0.020244 |
| Tirap | 1.542 | 0.053544 |
| Tnf | 22.872 | 0.001468 |
| Tnfsf11 | 19.8243 | 0.01125 |
| Tnfsf14 | 6.6063 | 0.003588 |
| Tnfsf4 | 1.2872 | 0.679304 |
| Traf6 | 3.1612 | 0.124326 |
| Txnip | -1.0346 | 0.770468 |
| Xiap | 2.6434 | 0.037012 |
| Gusb | -1.7573 | 0.094996 |
| Hprt | 1.1031 | 0.094868 |
| Hsp90ab1 | 1.5931 | 0.087613 |
| Gapdh | -31.1674 | 0.373903 |
| Actb | 143.7473 | 0.043633 |

**Supplementary Table S2: Gene expression analysis between 24 h *T. cruzi* infected (Tc 1 h condition) or uninfected macrophages (CTR condition).** Fold change and p-values associated with each gene analyzed in the PCR array. Genes with higher expression (fold change value ≥ 2) in Tc 1 h condition with respect to CTR condition are highlighted in red. In blue are highlighted those genes with lower expression (fold change value ≤ -2). Changes in gene expression associated with p-value lower than 0.05 are highlighted in red.
