# Supplementary material for: Therapeutic effects of sphingosine kinase inhibitor N,N-dimethylsphingosine (DMS) in experimental chronic Chagas disease cardiomyopathy
Source: Sci Rep. 2017 Jul 21;7:6171. doi: 10.1038/s41598-017-06275-z (PMC5522404; doi:10.1038/s41598-017-06275-z)
Supplement: Supplementary file 4 — Supplementary Table S3 [file 41598_2017_6275_MOESM4_ESM.doc]

| **Gene symbol** | **Fold change** | **p-value** |
| --- | --- | --- |
| Aim2 | -1.4426 | 0.367028 |
| Bcl2 | -1.3858 | 0.445976 |
| Bcl2l1 | -3.1491 | 0.211546 |
| Birc2 | 2.4155 | 0.067046 |
| Birc3 | 6.4649 | 0.000004 |
| Card6 | -1.0241 | 0.421448 |
| Casp1 | -1.289 | 0.383111 |
| Casp12 | -1.6693 | 0.276224 |
| Casp8 | -1.468 | 0.30716 |
| Ccl12 | -1.0379 | 0.26379 |
| Ccl5 | 2.0769 | 0.343272 |
| Ccl7 | 33.1482 | 0.000001 |
| Cd40lg | 1.0243 | 0.740297 |
| Cflar | 9.5208 | 0.000008 |
| Chuk | 1.5976 | 0.070798 |
| Ciita | -8.5334 | 0.103538 |
| Ctsb | -11.7573 | 0.005941 |
| Cxcl1 | 79.793 | 0.001702 |
| Cxcl3 | 10036.5554 | 0.000271 |
| Fadd | 2.0859 | 0.095279 |
| Hsp90aa1 | 8.4356 | 0.000377 |
| Hsp90ab1 | 1.245 | 0.21691 |
| Hsp90b1 | 4.3444 | 0.000831 |
| Ifnb1 | 28.1881 | 0.000089 |
| Ifng | 11.7767 | 0.007202 |
| Ikbkb | 3.4826 | 0.005527 |
| Ikbkg | 36.8658 | 0.000273 |
| Il12a | 357.1379 | 0.000112 |
| Il12b | 4.5348 | 0.047475 |
| Il18 | 2.3565 | 0.000749 |
| Il1b | 349.1325 | 0.00005 |
| Il33 | 102.0753 | 0 |
| Il6 | 3533.2955 | 0.000017 |
| Irak1 | 1.7994 | 0.067593 |
| Irf1 | 1.9924 | 0.317959 |
| Irf2 | -1.0295 | 0.777006 |
| Irf3 | 2.2791 | 0.226401 |
| Map3k7 | 4.4434 | 0.001143 |
| Tab1 | 1.9483 | 0.182748 |
| Tab2 | 2.0529 | 0.001189 |
| Mapk1 | 2.9786 | 0.001923 |
| Mapk11 | 3.2816 | 0.055182 |
| Mapk12 | 3.1855 | 0.524576 |
| Mapk13 | 2.3442 | 0.087328 |
| Mapk3 | 2.2838 | 0.050396 |
| Mapk8 | 11.0235 | 0.000047 |
| Mapk9 | 2.1634 | 0.065781 |
| Mefv | 19.1579 | 0.005503 |
| Myd88 | 3.9276 | 0.020573 |
| Naip1 | 2.5721 | 0.001094 |
| Naip5 | -1.4065 | 0.352698 |
| Nfkb1 | 5.1814 | 0.000068 |
| Nfkbia | 5.2772 | 0.00007 |
| Nfkbib | 10.8161 | 0.000757 |
| Nlrc4 | 18.5767 | 0.000389 |
| Nlrc5 | 3.4844 | 0.025084 |
| Nlrp1a | 3.5113 | 0.143553 |
| Nlrp3 | 9.8143 | 0.000032 |
| Nlrp4b | 1.4798 | 0.59583 |
| Nlrp4e | 1.4798 | 0.59583 |
| Nlrp5 | 1.4798 | 0.59583 |
| Nlrp6 | 1.4798 | 0.59583 |
| Nlrp9b | 1.4798 | 0.59583 |
| Nlrx1 | 1.1035 | 0.856183 |
| Nod2 | 6.8254 | 0.002196 |
| P2rx7 | 1.78 | 0.188511 |
| Panx1 | -1.111 | 0.464705 |
| Pea15a | -2.3451 | 0.175247 |
| Pstpip1 | -2.9149 | 0.128207 |
| Ptgs2 | 532.7361 | 0.000069 |
| Pycard | 1.437 | 0.792927 |
| Mok | 2.436 | 0.080434 |
| Rela | 4.5134 | 0.00301 |
| Ripk2 | 6.1795 | 0.000316 |
| Sugt1 | 5.6694 | 0.002545 |
| Tirap | 2.1454 | 0.005794 |
| Tnf | 24.2276 | 0.000001 |
| Tnfsf11 | 16.7437 | 0.002381 |
| Tnfsf14 | 5.422 | 0.00947 |
| Tnfsf4 | 2.6554 | 0.113014 |
| Traf6 | 5.1228 | 0.004847 |
| Txnip | -1.4182 | 0.049299 |
| Xiap | 3.8481 | 0.001186 |
| Gusb | -1.4784 | 0.160218 |
| Hprt | 1.0638 | 0.231563 |
| Hsp90ab1 | 1.3898 | 0.180263 |
| Gapdh | -18.7971 | 0.373904 |
| Actb | 192.8552 | 0.002188 |

**Supplementary Table S3: Gene expression analysis between DMS treatment for 1 h in *T. cruzi* infected macrophages (Tc + DMS 1 h condition) with respect to uninfected macrophages (CTR condition).** Fold change and p-values associated with each gene analyzed in the PCR array. Genes with higher expression (fold change value ≥ 2) in Tc + DMS 1 h condition with respect to CTR condition are highlighted in red. In blue are highlighted those genes with lower expression (fold change value ≤ -2). Changes in gene expression associated with p-value lower than 0.05 are highlighted in red.
