# Supplementary material for: Therapeutic effects of sphingosine kinase inhibitor N,N-dimethylsphingosine (DMS) in experimental chronic Chagas disease cardiomyopathy
Source: Sci Rep. 2017 Jul 21;7:6171. doi: 10.1038/s41598-017-06275-z (PMC5522404; doi:10.1038/s41598-017-06275-z)
Supplement: Supplementary file 5 — Supplementary Table S4 [file 41598_2017_6275_MOESM5_ESM.doc]

| **Gene symbol** | **Fold change** | **p-value** |
| --- | --- | --- |
| Aim2 | -2.6499 | 0.157032 |
| Bcl2 | 2.1045 | 0.366951 |
| Bcl2l1 | 1.3306 | 0.990647 |
| Birc2 | 1.7183 | 0.406691 |
| Birc3 | 2.5438 | 0.003086 |
| Card6 | -1.5271 | 0.400903 |
| Casp1 | 1.1312 | 0.893556 |
| Casp12 | -1.2805 | 0.833631 |
| Casp8 | 1.0779 | 0.925837 |
| Ccl12 | 17.2523 | 0.07501 |
| Ccl5 | 10.2092 | 0.090648 |
| Ccl7 | 5.07 | 0.01103 |
| Cd40lg | 1.1883 | 0.801577 |
| Cflar | 2.859 | 0.024169 |
| Chuk | -1.6645 | 0.060045 |
| Ciita | 1.3888 | 0.83868 |
| Ctsb | -3.4928 | 0.014083 |
| Cxcl1 | 3.3956 | 0.074636 |
| Cxcl3 | 147.7595 | 0.003663 |
| Fadd | -2.0958 | 0.23102 |
| Hsp90aa1 | 1.9358 | 0.355878 |
| Hsp90ab1 | -1.0503 | 0.708495 |
| Hsp90b1 | 1.7452 | 0.137164 |
| Ifnb1 | 18.3695 | 0.000832 |
| Ifng | 35.4161 | 0.000114 |
| Ikbkb | 1.8691 | 0.148527 |
| Ikbkg | 3.5084 | 0.074602 |
| Il12a | 42.5877 | 0.009779 |
| Il12b | 6.4127 | 0.067407 |
| Il18 | 1.858 | 0.039101 |
| Il1b | 8.7732 | 0.015531 |
| Il33 | 11.9183 | 0.031725 |
| Il6 | 113.2813 | 0.021593 |
| Irak1 | 1.1378 | 0.818896 |
| Irf1 | 4.4016 | 0.034173 |
| Irf2 | 1.0919 | 0.423149 |
| Irf3 | -1.062 | 0.634377 |
| Map3k7 | 1.4084 | 0.523795 |
| Tab1 | -1.0656 | 0.794888 |
| Tab2 | 1.3262 | 0.264051 |
| Mapk1 | 1.2987 | 0.471066 |
| Mapk11 | 1.9644 | 0.450082 |
| Mapk12 | 1.1585 | 0.583229 |
| Mapk13 | 2.3241 | 0.198876 |
| Mapk3 | 1.702 | 0.253457 |
| Mapk8 | 1.6785 | 0.413121 |
| Mapk9 | 2.2618 | 0.127935 |
| Mefv | 5.6786 | 0.268754 |
| Myd88 | 3.7586 | 0.05481 |
| Naip1 | 1.358 | 0.260366 |
| Naip5 | -1.1488 | 0.564805 |
| Nfkb1 | 1.6974 | 0.046902 |
| Nfkbia | 3.2831 | 0.003719 |
| Nfkbib | 2.3317 | 0.037868 |
| Nlrc4 | 5.6358 | 0.09925 |
| Nlrc5 | 3.6991 | 0.053525 |
| Nlrp1a | 5.1688 | 0.027584 |
| Nlrp3 | 1.7209 | 0.098958 |
| Nlrp4b | 1.5569 | 0.518294 |
| Nlrp4e | 1.5569 | 0.518294 |
| Nlrp5 | 1.5569 | 0.518294 |
| Nlrp6 | 1.5569 | 0.518294 |
| Nlrp9b | 1.5569 | 0.518294 |
| Nlrx1 | 2.0371 | 0.307313 |
| Nod2 | 2.7207 | 0.053432 |
| P2rx7 | 1.2265 | 0.869498 |
| Panx1 | 1.4698 | 0.077425 |
| Pea15a | -2.6348 | 0.169642 |
| Pstpip1 | 2.175 | 0.09912 |
| Ptgs2 | 7.9408 | 0.015234 |
| Pycard | 1.8437 | 0.41814 |
| Mok | 1.7494 | 0.338838 |
| Rela | 2.3119 | 0.101336 |
| Ripk2 | 2.4972 | 0.000859 |
| Sugt1 | 1.6961 | 0.330879 |
| Tirap | 1.1484 | 0.478037 |
| Tnf | 6.0836 | 0.003242 |
| Tnfsf11 | 13.3717 | 0.001634 |
| Tnfsf14 | 1.4868 | 0.96489 |
| Tnfsf4 | 1.459 | 0.492761 |
| Traf6 | 3.0644 | 0.085421 |
| Txnip | -1.3721 | 0.096632 |
| Xiap | 1.2557 | 0.668591 |
| Gusb | -1.0608 | 0.663276 |
| Hprt | -1.0362 | 0.462713 |
| Hsp90ab1 | 1.0992 | 0.788647 |
| Gapdh | -33.4388 | 0.373902 |
| Actb | 125.5043 | 0.030392 |

**Supplementary Table S4: Gene expression analysis between 48 h *T. cruzi*-infected (Tc 24 h condition) or uninfected macrophages (CTR condition).** Fold change and p-values associated with each gene analyzed in the PCR array. Genes with higher expression (fold change value ≥ 2) in Tc 24 h condition with respect to CTR condition are highlighted in red. In blue are highlighted those genes with lower expression (fold change value ≤ -2). Changes in gene expression associated with p-value lower than 0.05 are highlighted in red.
