# Supplementary material for: Therapeutic effects of sphingosine kinase inhibitor N,N-dimethylsphingosine (DMS) in experimental chronic Chagas disease cardiomyopathy
Source: Sci Rep. 2017 Jul 21;7:6171. doi: 10.1038/s41598-017-06275-z (PMC5522404; doi:10.1038/s41598-017-06275-z)
Supplement: Supplementary file 6 — Supplementary Table S5 [file 41598_2017_6275_MOESM6_ESM.doc]

| **Gene symbol** | **Fold change** | **p-value** |
| --- | --- | --- |
| Aim2 | 1.1651 | 0.72541 |
| Bcl2 | 2.0649 | 0.407369 |
| Bcl2l1 | 1.6379 | 0.696732 |
| Birc2 | 1.8739 | 0.341315 |
| Birc3 | 2.7761 | 0.000448 |
| Card6 | -1.169 | 0.495176 |
| Casp1 | 1.3897 | 0.433473 |
| Casp12 | 3.0588 | 0.311212 |
| Casp8 | 1.6551 | 0.438834 |
| Ccl12 | 20.5336 | 0.026903 |
| Ccl5 | 8.1825 | 0.229037 |
| Ccl7 | 5.0894 | 0.005319 |
| Cd40lg | 3.8976 | 0.373671 |
| Cflar | 2.8185 | 0.012627 |
| Chuk | -1.3536 | 0.281632 |
| Ciita | 2.6555 | 0.294037 |
| Ctsb | -2.2401 | 0.040715 |
| Cxcl1 | 2.734 | 0.105099 |
| Cxcl3 | 146.3651 | 0.000444 |
| Fadd | 1.6016 | 0.40928 |
| Hsp90aa1 | 1.6215 | 0.590827 |
| Hsp90ab1 | 1.0852 | 0.711896 |
| Hsp90b1 | 1.6892 | 0.141403 |
| Ifnb1 | 28.1276 | 0.004367 |
| Ifng | 37.4181 | 0.001139 |
| Ikbkb | 1.5471 | 0.478044 |
| Ikbkg | 2.6818 | 0.230921 |
| Il12a | 46.0227 | 0.011904 |
| Il12b | 10.9787 | 0.232906 |
| Il18 | 1.7852 | 0.154451 |
| Il1b | 6.9614 | 0.030368 |
| Il33 | 8.8668 | 0.115945 |
| Il6 | 90.149 | 0.080986 |
| Irak1 | 1.2413 | 0.589482 |
| Irf1 | 4.0595 | 0.0479 |
| Irf2 | 1.1095 | 0.477916 |
| Irf3 | 1.5688 | 0.451817 |
| Map3k7 | 1.2683 | 0.697734 |
| Tab1 | 1.7067 | 0.449749 |
| Tab2 | 1.2532 | 0.308063 |
| Mapk1 | 1.2124 | 0.638145 |
| Mapk11 | 3.5974 | 0.322909 |
| Mapk12 | 3.0131 | 0.458983 |
| Mapk13 | 3.6904 | 0.360797 |
| Mapk3 | 1.5305 | 0.386084 |
| Mapk8 | 1.9249 | 0.364341 |
| Mapk9 | 2.2634 | 0.259141 |
| Mefv | 7.6856 | 0.248863 |
| Myd88 | 3.2405 | 0.088896 |
| Naip1 | 1.6806 | 0.132086 |
| Naip5 | 1.013 | 0.940112 |
| Nfkb1 | 1.4859 | 0.30596 |
| Nfkbia | 2.7243 | 0.004986 |
| Nfkbib | 5.0477 | 0.261464 |
| Nlrc4 | 4.6759 | 0.297131 |
| Nlrc5 | 4.0458 | 0.119126 |
| Nlrp1a | 4.0705 | 0.347244 |
| Nlrp3 | 2.0063 | 0.088648 |
| Nlrp4b | 3.0121 | 0.383095 |
| Nlrp4e | 3.4229 | 0.379884 |
| Nlrp5 | 3.4401 | 0.379786 |
| Nlrp6 | 3.4928 | 0.379499 |
| Nlrp9b | 4.1539 | 0.377105 |
| Nlrx1 | 3.9477 | 0.212104 |
| Nod2 | 3.1042 | 0.069978 |
| P2rx7 | 1.393 | 0.588593 |
| Panx1 | 1.6097 | 0.045137 |
| Pea15a | -2.4835 | 0.171696 |
| Pstpip1 | 2.0537 | 0.121112 |
| Ptgs2 | 7.183 | 0.0069 |
| Pycard | 1.8358 | 0.435078 |
| Mok | 3.1072 | 0.381106 |
| Rela | 1.9634 | 0.281379 |
| Ripk2 | 2.3505 | 0.008687 |
| Sugt1 | 1.6556 | 0.463003 |
| Tirap | -1.0795 | 0.802465 |
| Tnf | 5.278 | 0.008935 |
| Tnfsf11 | 15.9123 | 0.019615 |
| Tnfsf14 | 2.2305 | 0.417111 |
| Tnfsf4 | 2.9561 | 0.383375 |
| Traf6 | 2.6507 | 0.300827 |
| Txnip | -1.3529 | 0.150573 |
| Xiap | -1.1725 | 0.528598 |
| Gusb | -1.0544 | 0.679054 |
| Hprt | -1.0152 | 0.711991 |
| Hsp90ab1 | 1.0704 | 0.886148 |
| Gapdh | -41.7168 | 0.373902 |
| Actb | 100.8013 | 0.04038 |

**Supplementary Table S5: Gene expression analysis between DMS treatment for 24 h in *T. cruzi*-infected macrophages (Tc + DMS 24 h condition) with respect to uninfected macrophages (CTR condition).** Fold change and p-values associated with each gene analyzed in the PCR array. Genes with higher expression (fold change value ≥ 2) in Tc + DMS 24 h condition with respect to CTR condition are highlighted in red. In blue are highlighted those genes with lower expression (fold change value ≤ -2). Changes in gene expression associated with p-value lower than 0.05 are highlighted in red.
