# Supplementary material for: Therapeutic effects of sphingosine kinase inhibitor N,N-dimethylsphingosine (DMS) in experimental chronic Chagas disease cardiomyopathy
Source: Sci Rep. 2017 Jul 21;7:6171. doi: 10.1038/s41598-017-06275-z (PMC5522404; doi:10.1038/s41598-017-06275-z)
Supplement: Supplementary file 7 — Supplementary Table S6 [file 41598_2017_6275_MOESM7_ESM.doc]

| **Gene symbol** | **Fold change** | **p-value** |
| --- | --- | --- |
| Aim2 | -1.3444 | 0.271487 |
| Bcl2 | 1.2596 | 0.532463 |
| Bcl2l1 | 1.5345 | 0.145808 |
| Birc2 | 1.3254 | 0.14494 |
| Birc3 | -1.2693 | 0.107699 |
| Card6 | 2.2131 | 0.096533 |
| Casp1 | -1.3513 | 0.233844 |
| Casp12 | 1.4308 | 0.153724 |
| Casp8 | -1.1314 | 0.334293 |
| Ccl12 | -1.7678 | 0.132089 |
| Ccl5 | -1.4452 | 0.044923 |
| Ccl7 | -1.3872 | 0.185673 |
| Cd40lg | 2.0527 | 0.063317 |
| Cflar | 1.195 | 0.082884 |
| Chuk | -1.351 | 0.205708 |
| Ciita | 1.2366 | 0.408261 |
| Ctsb | -1.4584 | 0.247879 |
| Cxcl1 | -1.5522 | 0.070726 |
| Cxcl3 | -1.6436 | 0.136052 |
| Fadd | 1.6218 | 0.181863 |
| Hsp90aa1 | 1.1002 | 0.354508 |
| Hsp90ab1 | -1.3935 | 0.18954 |
| Hsp90b1 | -1.1958 | 0.093745 |
| Ifnb1 | -1.586 | 0.232226 |
| Ifng | -1.1258 | 0.582897 |
| Ikbkb | 1.8173 | 0.028309 |
| Ikbkg | 2.133 | 0.028963 |
| Il12a | 1.094 | 0.470158 |
| Il12b | 1.5772 | 0.356191 |
| Il18 | -1.2139 | 0.034952 |
| Il1b | -1.5151 | 0.076464 |
| Il33 | 1.0781 | 0.536039 |
| Il6 | -1.647 | 0.000255 |
| Irak1 | -1.2384 | 0.142982 |
| Irf1 | 1.3055 | 0.141087 |
| Irf2 | -1.2114 | 0.229736 |
| Irf3 | 1.8311 | 0.002777 |
| Map3k7 | 1.0048 | 0.930266 |
| Tab1 | 2.2416 | 0.017873 |
| Tab2 | 1.2892 | 0.021897 |
| Mapk1 | 1.1476 | 0.021119 |
| Mapk11 | 1.0028 | 0.977305 |
| Mapk12 | 1.2528 | 0.062859 |
| Mapk13 | -1.3499 | 0.291285 |
| Mapk3 | 1.1994 | 0.113728 |
| Mapk8 | -1.0271 | 0.69255 |
| Mapk9 | 1.1931 | 0.238244 |
| Mefv | 1.487 | 0.119867 |
| Myd88 | 1.73 | 0.197865 |
| Naip1 | -1.0447 | 0.393002 |
| Naip5 | 1.0353 | 0.83972 |
| Nfkb1 | 1.1201 | 0.250214 |
| Nfkbia | -1.199 | 0.0146 |
| Nfkbib | -1.1726 | 0.222149 |
| Nlrc4 | 1.6271 | 0.099556 |
| Nlrc5 | 3.3619 | 0.011742 |
| Nlrp1a | -1.0389 | 0.679932 |
| Nlrp3 | -1.3195 | 0.140481 |
| Nlrp4b | 2.868 | 0.074813 |
| Nlrp4e | 2.868 | 0.074813 |
| Nlrp5 | 2.868 | 0.074813 |
| Nlrp6 | 2.868 | 0.074813 |
| Nlrp9b | 2.868 | 0.074813 |
| Nlrx1 | 2.63 | 0.046743 |
| Nod2 | 1.8472 | 0.034353 |
| P2rx7 | 1.4605 | 0.015858 |
| Panx1 | -1.367 | 0.093719 |
| Pea15a | -1.5146 | 0.175976 |
| Pstpip1 | 1.3128 | 0.067096 |
| Ptgs2 | 1.1658 | 0.270067 |
| Pycard | -1.0475 | 0.312351 |
| Mok | -1.1868 | 0.451132 |
| Rela | 1.7222 | 0.040281 |
| Ripk2 | -1.2466 | 0.066469 |
| Sugt1 | 1.3613 | 0.056631 |
| Tirap | 1.1857 | 0.163488 |
| Tnf | -1.1078 | 0.017673 |
| Tnfsf11 | -1.3893 | 0.142846 |
| Tnfsf14 | -1.4297 | 0.163931 |
| Tnfsf4 | 1.758 | 0.153927 |
| Traf6 | 1.381 | 0.148062 |
| Txnip | -1.6085 | 0.090855 |
| Xiap | 1.2406 | 0.041207 |
| Gusb | 1.013 | 0.815696 |
| Hprt | -1.2168 | 0.159266 |
| Hsp90ab1 | -1.3451 | 0.218779 |
| Gapdh | 1.4131 | 0.051386 |
| Actb | 1.1434 | 0.508436 |

**Supplementary Table S6: Gene expression analysis between *T. cruzi*-infected macrophages with (TC + DMS 1 h condition) or without (Tc 1 h condition) 1 h DMS treatment.** Fold change and p-values associated with each gene analyzed in the PCR array. Genes with higher expression (fold change value ≥ 2) in Tc + DMS 1 h condition with respect to Tc 1 h condition are highlighted in red. In blue are highlighted those genes with lower expression (fold change value ≤ -2). Changes in gene expression associated with p-value lower than 0.05 are highlighted in red.
