# Supplementary material for: Therapeutic effects of sphingosine kinase inhibitor N,N-dimethylsphingosine (DMS) in experimental chronic Chagas disease cardiomyopathy
Source: Sci Rep. 2017 Jul 21;7:6171. doi: 10.1038/s41598-017-06275-z (PMC5522404; doi:10.1038/s41598-017-06275-z)
Supplement: Supplementary file 8 — Supplementary Table S7 [file 41598_2017_6275_MOESM8_ESM.doc]

| **Gene symbol** | **Fold change** | | **p-value** |
| --- | --- | --- | --- |
| Aim2 | | 3.3717 | 0.145681 |
| Bcl2 | | 1.0715 | 0.54706 |
| Bcl2l1 | | 1.3442 | 0.105955 |
| Birc2 | | 1.1909 | 0.601991 |
| Birc3 | | 1.1918 | 0.201682 |
| Card6 | | 1.4265 | 0.449808 |
| Casp1 | | 1.3415 | 0.239891 |
| Casp12 | | 4.2775 | 0.287485 |
| Casp8 | | 1.6769 | 0.354102 |
| Ccl12 | | 1.2998 | 0.38745 |
| Ccl5 | | -1.1425 | 0.402878 |
| Ccl7 | | 1.0963 | 0.804026 |
| Cd40lg | | 3.582 | 0.376773 |
| Cflar | | 1.0766 | 0.543902 |
| Chuk | | 1.3429 | 0.226987 |
| Ciita | | 2.088 | 0.078971 |
| Ctsb | | 1.7027 | 0.127331 |
| Cxcl1 | | -1.1373 | 0.496688 |
| Cxcl3 | | 1.0818 | 0.845135 |
| Fadd | | 3.6656 | 0.37896 |
| Hsp90aa1 | | -1.0932 | 0.525243 |
| Hsp90ab1 | | 1.2448 | 0.229906 |
| Hsp90b1 | | 1.057 | 0.585963 |
| Ifnb1 | | 1.6722 | 0.041903 |
| Ifng | | 1.1538 | 0.214309 |
| Ikbkb | | -1.1063 | 0.906509 |
| Ikbkg | | -1.1979 | 0.811212 |
| Il12a | | 1.1802 | 0.565001 |
| Il12b | | 1.8696 | 0.378712 |
| Il18 | | 1.0493 | 0.789007 |
| Il1b | | -1.154 | 0.350979 |
| Il33 | | -1.2308 | 0.733348 |
| Il6 | | -1.1507 | 0.821772 |
| Irak1 | | 1.1914 | 0.323339 |
| Irf1 | | 1.0072 | 0.987735 |
| Irf2 | | 1.1097 | 0.177396 |
| Irf3 | | 1.8195 | 0.412403 |
| Map3k7 | | -1.0169 | 0.985852 |
| Tab1 | | 1.9861 | 0.367098 |
| Tab2 | | 1.0319 | 0.840635 |
| Mapk1 | | 1.0196 | 0.816212 |
| Mapk11 | | 1.9999 | 0.350821 |
| Mapk12 | | 2.8404 | 0.357568 |
| Mapk13 | | 1.734 | 0.432456 |
| Mapk3 | | -1.0183 | 0.999113 |
| Mapk8 | | 1.2524 | 0.507349 |
| Mapk9 | | 1.0928 | 0.675348 |
| Mefv | | 1.4781 | 0.438789 |
| Myd88 | | -1.0621 | 0.730644 |
| Naip1 | | 1.3514 | 0.209912 |
| Naip5 | | 1.2708 | 0.281129 |
| Nfkb1 | | -1.046 | 0.980972 |
| Nfkbia | | -1.1035 | 0.306872 |
| Nfkbib | | 2.3641 | 0.311226 |
| Nlrc4 | | -1.1037 | 0.839955 |
| Nlrc5 | | 1.1944 | 0.558422 |
| Nlrp1a | | -1.1628 | 0.526666 |
| Nlrp3 | | 1.2732 | 0.369518 |
| Nlrp4b | | 2.1129 | 0.408317 |
| Nlrp4e | | 2.401 | 0.396373 |
| Nlrp5 | | 2.4131 | 0.396007 |
| Nlrp6 | | 2.4501 | 0.394936 |
| Nlrp9b | | 2.9138 | 0.385981 |
| Nlrx1 | | 2.1163 | 0.242707 |
| Nod2 | | 1.246 | 0.467638 |
| P2rx7 | | 1.2404 | 0.060179 |
| Panx1 | | 1.196 | 0.402588 |
| Pea15a | | 1.1586 | 0.654191 |
| Pstpip1 | | 1.0312 | 0.835837 |
| Ptgs2 | | -1.0123 | 0.869543 |
| Pycard | | 1.0874 | 0.720446 |
| Mok | | 1.9396 | 0.41394 |
| Rela | | -1.0782 | 0.851188 |
| Ripk2 | | 1.0279 | 0.718178 |
| Sugt1 | | 1.066 | 0.671814 |
| Tirap | | -1.1352 | 0.924422 |
| Tnf | | -1.0554 | 0.828882 |
| Tnfsf11 | | 1.2996 | 0.229781 |
| Tnfsf14 | | 1.6384 | 0.15939 |
| Tnfsf4 | | 2.2126 | 0.400044 |
| Traf6 | | -1.0586 | 0.894816 |
| Txnip | | 1.1076 | 0.603624 |
| Xiap | | -1.3482 | 0.229508 |
| Gusb | | 1.0987 | 0.383619 |
| Hprt | | 1.1146 | 0.293479 |
| Hsp90ab1 | | 1.0635 | 0.680677 |
| Gapdh | | -1.1424 | 0.652389 |
| Actb | | -1.1401 | 0.304543 |

**Supplementary Table S7: Gene expression analysis between *T. cruzi*-infected macrophages with (Tc + DMS 24 h condition) or without (Tc 24 h condition) 24 h DMS treatment.** Fold change and p-values associated with each gene analyzed in the PCR array. Genes with higher expression (fold change value ≥ 2) in Tc + DMS 24 h condition with respect to Tc 24 h condition are highlighted in red. In blue are highlighted those genes with lower expression (fold change value ≤ -2). Changes in gene expression associated with p-value lower than 0.05 are highlighted in red.
